# A moral trade-off system produces intuitive judgments that are rational and coherent and strike a balance between conflicting moral values

**DOI:** 10.1073/pnas.2214005119

**Published:** 2022-10-10

**Authors:** Ricardo Andrés Guzmán, María Teresa Barbato, Daniel Sznycer, Leda Cosmides

**Affiliations:** ^a^Centro de Investigación en Complejidad Social, Facultad de Gobierno, Universidad del Desarrollo, Santiago 7610658, Chile;; ^b^Department of Psychology, University of Montreal, Montreal, QC, Canada H3C 3J7;; ^c^Oklahoma Center for Evolutionary Analysis, Department of Psychology, Oklahoma State University, Stillwater, OK 74078-3064;; ^d^Center for Evolutionary Psychology, Department of Psychological & Brain Sciences, University of California, Santa Barbara, CA 93106-9660

**Keywords:** moral psychology, evolutionary psychology, moral dilemmas, judgment and decision-making, moral value pluralism

## Abstract

How does the mind make moral judgments when the only way to satisfy one moral value is to neglect another? Moral dilemmas posed a recurrent adaptive problem for ancestral hominins, whose cooperative social life created multiple responsibilities to others. For many dilemmas, striking a balance between two conflicting values (a compromise judgment) would have promoted fitness better than neglecting one value to fully satisfy the other (an extreme judgment). We propose that natural selection favored the evolution of a cognitive system designed for making trade-offs between conflicting moral values. Its nonconscious computations respond to dilemmas by constructing “rightness functions”: temporary representations specific to the situation at hand. A rightness function represents, in compact form, an ordering of all the solutions that the mind can conceive of (whether feasible or not) in terms of moral rightness. An optimizing algorithm selects, among the feasible solutions, one with the highest level of rightness. The moral trade-off system hypothesis makes various novel predictions: People make compromise judgments, judgments respond to incentives, judgments respect the axioms of rational choice, and judgments respond coherently to morally relevant variables (such as willingness, fairness, and reciprocity). We successfully tested these predictions using a new trolley-like dilemma. This dilemma has two original features: It admits both extreme and compromise judgments, and it allows incentives—in this case, the human cost of saving lives—to be varied systematically. No other existing model predicts the experimental results, which contradict an influential dual-process model.

## Significance

Intuitions about right and wrong clash in moral dilemmas. We report evidence that dilemmas activate a moral trade-off system: a cognitive system that is well designed for making trade-offs between conflicting moral values. When asked which option for resolving a dilemma is morally right, many people made compromise judgments, which strike a balance between conflicting moral values by partially satisfying both. Furthermore, their moral judgments satisfied a demanding standard of rational choice: the Generalized Axiom of Revealed Preferences. Deliberative reasoning cannot explain these results, nor can a tug-of-war between emotion and reason. The results are the signature of a cognitive system that weighs competing moral considerations and chooses the solution that maximizes rightness.

Moral dilemmas are an inescapable aspect of the human condition because no single principle regulates judgments in all social interactions (1–4). Natural selection wrote different rulebooks for siblings, parents and offspring, cooperative partners, and coalitional allies, to mention a few. Different cognitive systems evolved for navigating each of these relationships, including ones specialized for helping kin (5), trading goods and favors (1, 6–8), pooling risk in foraging (9–11), and cooperating in groups (3, 12, 13). Each cognitive system is equipped with different concepts and inferential mechanisms, which generate moral intuitions tailored to its domain.

In many situations, moral intuitions collide. A situation in which your friend and sibling both need help may be represented by two distinct systems, each generating different intuitions about the right thing to do. Loyalty to your allies might harm an old friend. The day may be too short to fulfill your duties at both work and home.

When moral intuitions collide, solutions that strike a balance between conflicting moral values are usually possible. Has natural selection produced a cognitive system for making moral trade-offs like these?

Past studies cannot answer this question because they use moral dilemmas that force extreme judgments: ones that fully satisfy one moral value while neglecting others entirely (14, 15). Consider, by contrast, the following dilemma from warfare. It shares many properties with trolley dilemmas, without forcing an extreme judgment.

Two countries, A and B, have been at war for years (you are not a citizen of either country). The war was initiated by the rulers of B, against the will of the civilian population. Recently, the military equilibrium has broken, and it is certain that A will win. The question is how, when, and at what cost.

Country A has two strategies available: attacking the opposing army with conventional weapons and bombing the civilian population. They could use one, the other, or a combination of both. Bombing would demoralize country B: The more civilians are killed, the sooner B will surrender, and the fewer soldiers will die—about half from both sides, all forcibly drafted. Conventional fighting will minimize civilian casualties but maximize lives lost (all soldiers).

More precisely: If country A chooses not to bomb country B, then 6 million soldiers will die, but almost no civilians. If 4 million civilians are sacrificed in the bombings, B will surrender immediately, and almost no soldiers will die. And, if A chooses an intermediate solution, for every four civilians sacrificed, approximately six fewer soldiers will die.

How should country A end the war? What do you feel is morally right?

In moral psychology, “sacrifice 4 million civilians” is typically interpreted as a utilitarian response because it saves the most lives (all soldiers). The cost is inflicting maximum harm on civilians. “Do not sacrifice any civilians” is typically interpreted as a deontic response because it respects the principle of not harming bystanders (the civilians). The cost is maximizing the death toll. Both are extreme judgments, because they satisfy one moral value fully but the other not at all. Compromise judgments strike a balance between competing moral values by partially satisfying both. An intermediate solution, such as “sacrifice *x* civilians to save *y* soldiers,” is a compromise judgment: It spares some (but not all) civilians while saving more (but not the most) lives.*

**Fig. 1.**
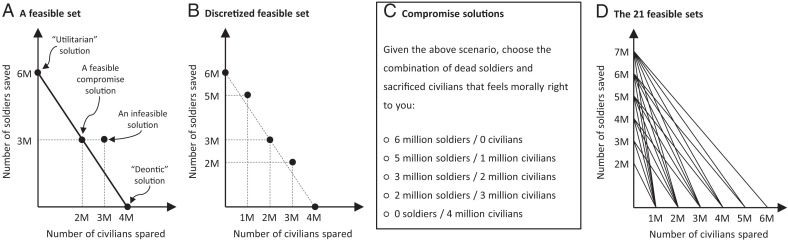
(*A*) Feasible set for the introductory example. Solutions are expressed in terms of “moral goods”: the lives of civilians and soldiers. (*B*) Discretized feasible set corresponding to that scenario. (*C*) Alternatives presented to subjects, corresponding to the solutions in the discretized feasible set (e.g., the alternative “0 sacrificed civilians and 6 million dead soldiers” corresponds to the feasible solution “4 million civilians spared and 0 soldiers saved”). Alternatives are expressed in terms of deaths, which are “moral bads.” (*D*) Feasible sets for the 21 scenarios of the war dilemma. Subjects responded to all 21 scenarios, for each willingness frame.

Each judgment is associated with a solution to this dilemma, expressed as a “bundle” of two moral goods: a number of civilians spared, denoted by *c*, and a number of soldiers saved, denoted by *s*. All solutions with c,s≥0 are conceivable, but not all are available. There is a feasibility constraint, defined by two conditions stated in the dilemma: s=6 million−1.5c, and c≤4 million. Fig. 1*A* represents these conditions graphically; all points on the line are feasible solutions for this dilemma. This feasible set includes the two extreme solutions—utilitarian and deontic—as well as all points in between: intermediate solutions, such as “2 million civilians spared and 3 million soldiers saved.” Solutions that fall above or below the line are not available to be chosen: You might prefer to spare 3 million civilians and 3 million soldiers, for example, but the feasible set does not include this solution.

The lifeways of our hunter-gatherer ancestors routinely created moral dilemmas (16–20). For many, a compromise judgment would have promoted fitness better than an extreme one. These situations should have selected for a cognitive system that is well designed for making trade-offs between conflicting moral values. The adaptive function of this moral trade-off system (MTS) would be to identify, among the available solutions, one that is most right. These judgments would inform (but not determine) behavior.

## The Moral Tradeoff System

### Four Design Features.

To accomplish its adaptive function well, an MTS requires features designed to solve four adaptive problems.

#### Feature 1.

The MTS should be able to produce the full spectrum of judgments: extreme ones and compromises.

Consider this dilemma. A forager fished all day, but his luck was bad. He returns to camp with a catch too small to feed his children and sick brother. The forager’s neighbor has been smoking fish for her grandchildren’s visit. He asks her for some, but she gives him far less than he requested. The forager feels it would be wrong to steal additional fish from his neighbor, but it would also be wrong to neglect his sick brother. Should he steal from her? If he does, how many fish should he take? The more he steals, the sooner his brother will recover, but the greater the harm to his neighbor.

The solution the forager experiences as most right could be to steal none, some, or all of her fish. The MTS should be capable of delivering any of those answers.

#### Feature 2.

Judgments should vary with incentives. These are variables that determine which solutions to the dilemma are feasible (that is, available to be chosen).

The solutions available to the forager depend on variables such as how much each fish he steals would improve his brother’s health and harm his neighbor. He may feel he should steal all of her fish if that would deliver his brother from the verge of death. If his brother has a cold, and some extra food would hasten his recovery by only a day, the forager may feel he should refrain from stealing altogether. If some extra food would significantly hasten his brother’s recovery without harming his neighbor too much, the forager may feel that he should steal some, but not all, of her fish.

#### Feature 3.

Judgments should vary with morally relevant variables, such as willingness, fairness, reciprocity, entitlement, merit, and honesty. The social cognitive systems activated by a dilemma determine which variables are morally relevant to its resolution.

Reciprocity, for instance, is morally relevant to the cognitive system that regulates social exchange (6). When the forager recalls that he helped his neighbor generously last week but she has been stingy in return, that cognitive system may infer that he is entitled to more fish than she gave him. A different “rulebook” regulates altruism toward kin: The forager will feel duty bound to care for his sick brother, even if his brother never reciprocates favors. The fact that the forager’s catch was small due to bad luck, not laziness, will be morally relevant to the cognitive system that regulates risk pool sharing: If the dilemma activates that system, it will deliver the intuition that he deserves help from his fellow fishers (21).

#### Feature 4.

The MTS should be able to weigh conflicting moral values and choose a solution that is most right, delivering that solution as an intuitive moral judgment.

The forager has a dilemma because his obligation to help his brother conflicts with his obligation to not harm his neighbor. His MTS should be able to strike a balance between these obligations, and choose the solution he feels is most right. Suppose he feels it would be right to steal 3 of her 10 remaining fish—a solution that would reduce (but not minimize) his brother’s recovery time, without severely harming his neighbor. This intuitive moral judgment will serve as input to decisions about how to behave (22, 23), along with prudential factors (e.g., risk of retribution) and temptations (e.g., the extra calories he could consume by stealing more than three fish).

### How an MTS Should Work.

These four adaptive problems have parallels in rational choice theory, which formally analyzes trade-off decisions. We repurposed tools from rational choice for moral trade-offs, and used this “theory of the computation” [*sensu* Marr (24)] to develop a cognitive model of how an MTS should work.

Considering a decision problem activates one or more social cognitive systems. Each system reads the situation, makes its own inferences, and creates a morally laden mental representation of the situation. If the different representations are contradictory, the MTS is activated.

We propose the MTS is composed of three subsystems: MV, FS, and MAX.

The MV subsystem integrates moral values. It receives the morally laden representations as inputs, assigns weights to the conflicting moral goods, and constructs a rightness function on the fly. A rightness function is a temporary mental representation (a type of data structure), specific to the situation at hand. The function maps all solutions that the mind can conceive of onto a level of rightness.^†^ When comparing two or more solutions, the one with the highest level of rightness will feel most right.

A rightness function can be denoted as follows:

v(x,β):X→V⊆R,

where *v* is the function; **x** is a conceivable solution; *X* is the set of conceivable solutions; *V* is the set of levels of rightness (which are real numbers); and β is a vector of situation-specific parameters computed by MV, by operating on the morally laden representations of the situation. The function gives rise to a rightness order.

$$\begin{array}{l} \text{If}\;v(x_{1},\beta )>v(x_{2},\beta ),\;x_{1}\;\text{feels}\;\text{more}\;\text{right}\;\text{than}\;x_{2}.\\ \text{If}\;v(x_{1},\beta )=v(x_{2},\beta ),\;x_{1}\;\text{and}\;x_{2}\text{feel}\;\text{equally}\;\text{right}. \end{array}$$

The parameters in β modulate the way in which the rightness function ranks solutions. It includes the weights assigned to the two moral goods: the lives of civilians and soldiers in the war dilemma, and the welfare of his brother and neighbor in the forager’s dilemma. Context can affect these parameters: If the civilians had supported the war, their lives would probably weigh less. If the neighbor had been generous, her welfare would probably weigh more.

The MV subsystem and the cognitive systems that inform it are universal, but they are calibrated by the individual’s experiences. So, faced with the same situation, different people might generate different parameter values. In consequence, their moral judgments may differ.

Working in parallel to MV, the FS (for feasible set) subsystem constructs a feasible set that captures the incentives of the dilemma. A feasible set is a mental representation of the solutions that the MTS perceives as available.

Subsequently, the MAX subsystem maximizes the rightness function, given the constraints posed by the feasible set. The result is an optimal solution $x^{*}$. MAX outputs the intuitive judgment “solution $x^{*}$ is the most right.”

When this process ends, the rightness function and feasible set are erased or fade from memory.

MV, FS, and MAX operate nonconsciously: Their computations are not performed via deliberative reasoning. The MTS operates like the visual system: Its final products are objects of awareness, but its computations are not. Just as we “see” objects, we “feel” that some options are more right than others. Like the sight of an object, the feeling “solution $x^{*}$ is most right” is a representation that can be read by many downstream systems, including those for deciding how to behave (25).

Even though the MTS operates nonconsciously, conscious deliberations can play a role in judgment. Arguments and reflection can change which social cognitive systems are activated by a dilemma and, therefore, the representations MV uses to compute β. This can affect intuitive judgments.

A cognitive system with this architecture is capable of producing compromise judgments (feature 1). It responds to incentives and morally relevant variables (features 2 and 3). And it assigns weights to competing moral goods, which allows it to choose a feasible solution that is most right (feature 4).

Rightness functions are a type of utility function, and maximizing a utility function produces choices that comply with the axioms of rational choice (26, 27). Alternative theories of moral judgment do not predict compromise judgments that respect the axioms of rational choice. Theories that attribute judgment to adaptive specializations are silent on these issues but consistent with them (3, 4, 8, 28–38). But rigid heuristics, inflexible emotions, or deliberative reasoning cannot explain rational judgments that include compromises. Indeed, a well-known dual-process model—one that invokes both inflexible emotions and deliberative reasoning—makes opposing predictions.

## A Competing Theory: Greene’s Model

Is it right to stop a runaway trolley by pushing a bystander onto the tracks, sacrificing his life to prevent the trolley from running over five workers? A prohibition against inflicting harm says no; this is considered a deontic judgment. A principle of maximizing aggregate welfare says yes; this is considered a utilitarian judgment. If making consistent deductions from a single normative principle is the standard for rationality (14, 15, 39), then human moral judgment falls short: Many people flip-flop between deontic and utilitarian judgments when the consequences of a trolley problem remain the same but other features of the situation change (14, 40, 41).

Moral flip-flopping is usually explained by a dual-process model (42). System 1 is composed of emotions, heuristics, and inferences, which produce moral intuitions. System 2 performs deliberative reasoning. Judgments can flip because the intuitions produced by system 1 can preempt, interfere with, or bias judgments reached by reasoning. These models vary greatly (3, 14, 36–38), but most assume that system 1 computations are automatic, nonconscious, effortless, and fast, whereas system 2 computations are controlled, conscious, effortful, and slow.

Greene’s dual-process model (14) makes three additional claims: 1) Emotions produce inflexible responses; 2) flexibility—responding to context by considering multiple factors—requires deliberative reasoning; and 3) utilitarian judgments are produced by reasoning, whereas deontic judgments are produced by emotions.

According to this model, we experience the trolley problem as a dilemma because “two [dissociable psychological] processes yield different answers to the same question” [ref. 15, p. 269]. When considering whether sacrificing the bystander to save five people is morally permissible, System 2, operating on emotionally neutral representations of the situation, does a cost–benefit analysis. Not pushing saves one life (the bystander), whereas pushing saves four (five workers minus one bystander), so System 2 outputs the utilitarian judgment: “Push the bystander.” But the prospect of pushing the bystander activates an “alarm bell” emotion, which issues an inflexible internal command: do not harm. That command translates into the deontic judgment: “Do not kill this innocent bystander!”

According to Greene and colleagues (14, 15), this prohibition against inflicting harm is “nonnegotiable”: It cannot be weighed against other values. Commands issued by the alarm bell are, by design, difficult to override, because their adaptive function is to prevent actions that disrupt cooperative relationships (14, 20).

Cushman and Greene (15) argue that nonnegotiability makes moral conflict fundamentally different from motivational conflict in other domains. “When motivational systems conflict, this conflict can often be negotiated by weighing the preferences against each other” (p. 276). “Intractable dilemmas arise when psychological systems produce outputs that are…non-negotiable because their outputs are processed *as absolute demands, rather than fungible preferences* [emphasis added].” As a result, the trolley problem is experienced as intractable: A deontic judgment will *feel* right, a utilitarian judgment will *seem* right, and subtle changes in context will cause people to flip between these two extremes (14, 40, 43). But no middle ground will ever feel right or seem right, because there is no psychological machinery for negotiating a compromise judgment.

Acknowledging that the nonnegotiability hypothesis is speculative, Cushman and Greene (15) say that “putting it to empirical test is an important matter for further research.” Here we test it—along with five novel MTS predictions.

## Testing the Nonnegotiability Hypothesis

Testing the nonnegotiability hypothesis requires a sacrificial moral dilemma that permits compromise judgments. The trolley problem and other standard dilemmas cannot be used, because they force subjects to choose extreme responses (e.g., push or do not push).

We used the war dilemma because it satisfies both requirements. It is sacrificial because increasing the number of survivors entails sacrificing bystanders: the civilians. It also permits compromise judgments.

The war dilemma has two additional features tailored to intensify a nonnegotiable demand. First, the dilemma has three of four characteristics, each of which makes inflicting harm less morally acceptable to people (41): The harm is inflicted on others, not self; it is instrumental, not a side-effect; and harming civilians is avoidable, not inevitable. Second, history tells us that the war dilemma would activate an alarm bell, if one exists. Bombing civilian targets caused moral outrage in World War II, leading to a revision of the Geneva Accords: They permit attacks on military targets, but they prohibit deliberate or indiscriminate attacks on civilians.

To illustrate how Greene’s dual-process model would handle the war dilemma, suppose that 6 million soldiers are at risk, and that each civilian sacrificed saves three soldiers.

If the prospect of sacrificing one bystander activates an alarm bell emotion, as Greene maintains, then the prospect of killing a civilian should too. The prohibition issued by this alarm—“Do not bomb civilians!”—should be experienced as a nonnegotiable demand. Because intermediate solutions kill civilians, they will not satisfy that demand. Only the deontic judgment, “spare all civilians (2 million) and save zero soldiers” should feel right.

System 2 would determine that sacrificing all 2 million civilians will maximize the number of survivors. Therefore, system 2 would conclude that “spare zero civilians and save all 6 million soldiers” is more right than other solutions.

Systems 1 and 2 would issue competing judgments for the war dilemma. But there is no psychological machinery that can weigh these moral preferences against each other to produce a compromise, because the prohibition against killing civilians is nonnegotiable. So people will always opt for an extreme solution, even when intermediate solutions are available. They will never make compromise judgments.

Contrasting predictions follow from the MTS model.

## Testing for a Moral Tradeoff System

The war dilemma is also designed to test features of the MTS. Warfare is a domain that was relevant during human evolution (19, 20) and activates multiple moral intuitions (3, 13, 44–46). Minimizing loss of life in warfare is often seen as a moral good; so is sparing innocent lives. Across societies, from hunter-gatherers to nation states, people make moral distinctions between warriors and those they protect—family and friends ancestrally, civilians now (44–46). So the evolved systems activated by the war dilemma are likely to produce conflicting moral intuitions, thereby activating the MTS.

### Compromise Moral Judgments.

A well-designed MTS should be able to produce extreme and compromise judgments (feature 1).

#### Prediction 1.

Compromise judgments will be common for the war dilemma.

Support for this prediction is evidence in favor of the MTS model and against the nonnegotiability hypothesis.

### Response to Incentives.

Judgments should respond to incentives (feature 2). We tested this feature by systematically varying the dilemma’s incentives over a succession of scenarios. These incentives are given by two parameters: *S*, the number of soldiers at risk of death, and *S*/*C*, the number of soldiers saved for each civilian sacrificed. In all scenarios, S/C>1, so that the number of deaths is always minimized when *C* civilians are sacrificed. The 21 feasible sets for these scenarios are depicted in Fig. 1*D*.

#### Prediction 2.

Subjects will respond to incentives by changing their judgments.

The “cost-effectiveness” of sacrificing one life has received attention in studies with standard dilemmas: Utilitarian judgments were more likely when sacrificing one life saved larger numbers of people. This makes sense on both evolutionary and philosophical grounds (23, 47).

#### Conjecture 1.

Subjects will say that a larger percentage of soldiers should be saved if *S*/*C* is greater, all else being equal.

### Response to Morally Relevant Variables.

Morally laden representations of the situation serve as input to the MTS. When a change in situation alters these representations, the MTS should be capable of responding with different judgments (feature 3).

In war, people’s willingness to participate is morally relevant to assigning responsibility, blame, and honor. The introductory example said the people at risk were unwilling participants: Civilians had “opposed the war,” and soldiers “were forcibly drafted.” If one of these variables in the vignette is changed to “civilians supported the war” or “soldiers volunteered,” the MV subsystem may construct a different rightness function. Maximizing a different rightness function is likely to shift judgments.

To study this effect, we created three different frames for the dilemma—BU: both unwilling, the baseline; CW: civilians willing, a variant in which the civilians supported the war (but soldiers remain unwilling); and SW: soldiers willing, a variant in which the soldiers volunteered (but civilians remain unwilling).

#### Prediction 3.

Between frames, subjects will make different judgments.

Each subject responded to the 21 scenarios twice, once to the BU frame and once to either CW or SW.

### Moral Coherence.

As a check that subjects are responding to willingness because of its *moral* relevance, we assessed whether their judgments shifted coherently with this variable. In the war dilemma, the logic of consent suggests that equal or greater harm should befall willing than unwilling participants (47), as follows.

#### Conjecture 2.

Holding *S* and *S*/*C* constant: Taking BU as a baseline, subjects will say an equal or greater number of civilians should be sacrificed in CW, and an equal or lesser number of civilians should be sacrificed in SW.

### Judgments Will Respect the Axioms of Rational Choice.

Rational choice models assume that an agent has a preference order. This allows the agent to compare any options it can conceive of. Preference orders vary with context, but they do not change when incentives change. Different agents can have different preferences, so, when facing the same problem, they might make different choices. Here, the MTS is an agent, and its preferences are moral: They rank solutions in terms of rightness.

Preference orders have several properties. Consistency states that the statements “**x** is at least as good as **y**” and “**y** is better than **x**” cannot both be true at the same time. Transitivity states that, if “**x** is at least as good as **y**” and “**y** is at least as good as **z**,” then “**x** is at least as good as **z**.” Rational choice theory assumes that, among all feasible options, the agent will choose the one that it most prefers, or one of the most preferred if there is a tie.

Revealed preference methods can be used to assess the consistency of a sequence of choices, as defined by the axioms of rational choice theory. Broadly speaking, the experimental strategy is to present a person with a sequence of problems that are identical in all respects except for the incentives. If she reveals, through her choices, that “**x** is at least as good as **y**,” she will not reveal through later choices that “**y** is better than **x**.” Each violation of this logical requirement is an inconsistency. The fewer the inconsistencies, the more rational the person’s choices.

We used a revealed preference method to test whether judgments are made by maximizing a rightness function.

#### Prediction 4.

Within each frame, a subject will reveal few inconsistencies.

If prediction 4 is confirmed even for subjects who chose compromises, that is strong evidence for an MTS and against the nonnegotiability hypothesis, which must view compromises as random errors.

### Rightness Functions Are Temporary.

If rightness functions are temporary mental representations, then the MTS can construct a different one for each frame, resulting in different judgments. We tested this hypothesis by examining subjects who changed their judgments with willingness.

#### Prediction 5.

Subjects who make different judgments between frames will reveal few inconsistencies within each frame.

## Empirical Investigation

1,745 subjects participated in two conditions each (order counterbalanced): one with the BU frame and another with a variant—either CW (*n* = 845) or SW (*n* = 900).

Each condition consisted of 21 scenarios of the war dilemma (order randomized) with the same frame but different incentives. Across scenarios, *S* varied from 2 million to 7 million deaths, *C* varied from 1 million to 6 million deaths, and *S*/*C* varied from 1.17 to 7 soldiers saved per civilian sacrificed. The values of *S*/*C* cover the range typical of trolley problems. Fig. 1*D* depicts the feasible sets of the 21 scenarios. All include extreme solutions (deontic and utilitarian). The scenarios were repeated across frames, so every subject encountered each scenario twice.

A scenario offers several alternatives for ending a war. An alternative consists of a number of civilians sacrificed and a number of soldiers killed. Fig. 1*C* shows the alternatives presented to the subjects in one scenario, while Fig. 1*B* depicts the discretized feasible set. Alternatives are bundles of moral bads (deaths), whereas the feasible solutions are bundles of moral goods (lives). To simplify choices, we rounded lives to the nearest million. This resulted in two to seven alternatives per scenario. For comparison to past studies, six scenarios offer only extreme solutions. Examples are shown in Table 1 (see *SI Appendix*, Table S1 for all 21).

**Table 1.** Four scenarios of the dilemma

No. soldiers at risk (*S*)	No. soldiers saved for each civilian sacrificed (*S* / *C*)	Alternatives (civilians sacrificed, soldiers dead)	Feasible set (civilians spared, soldiers saved)
4	2.00	(0, 4) (1, 2) (2, 0)	(2, 0) (1, 2) (0, 4)
6	2.00	(0, 6) (1, 2, 4) (3, 0)	(3, 0) (1, 2, 4) (0, 6)
7	3.50	(0, 7) (1, 4) (2, 0)	(2, 0) (1, 3) (0, 7)
7	1.75	(0, 7) (1–4, 6) (4, 0)	(4, 0) (1–3, 5) (0, 7)

Note: All quantities in millions of lives.

Since our hypothesis is about moral intuitions (not the ability to reason from a philosophical principle), subjects were encouraged to answer “what you feel is morally right, which may or may not be the same as what you think is morally right.” The full text of the instrument is provided in *SI Appendix*.

## Results and Discussion

### Compromise Judgments Were Common.

**Fig. 2.**
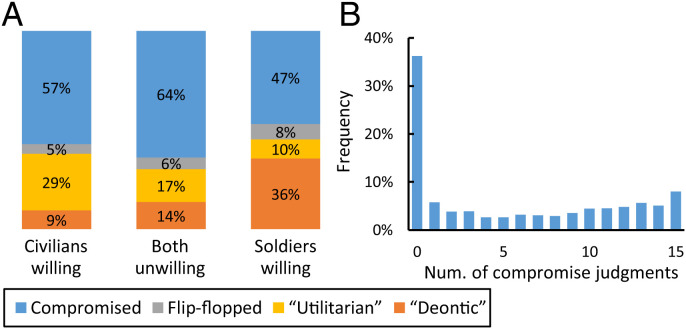
(*A*) The majority of subjects made compromise judgments. (*B*) Eighty-five percent of subjects who compromised made three or more compromise judgments. BU is pictured.

Prediction 1 was confirmed: 71% of subjects made compromise judgments in at least one condition. Fig. 2*A* shows the percent who made compromises in each frame. We will call these subjects “compromisers.” The figure also shows the percent of subjects who made the same extreme judgment 21 times and the percent who flip-flopped between extreme judgments. We will call these subjects “extreme responders” and “flip-floppers.”

The nonnegotiability hypothesis predicts that subjects always intend to make an extreme judgment, because they lack the cognitive capacity to negotiate deontic and utilitarian values. They could, however, make compromise judgments occasionally, due to “trembling hand” mistakes: clicking on an option other than intended by accident (due to a slip of the hand or lapse of attention). This could happen in the 15 scenarios with intermediate solutions.

As Fig. 2*B* shows, the distribution of compromise judgments is incompatible with a preference for extreme judgments, peppered with an occasional mistake. If hand trembling were the correct explanation, the “compromised” category would have been crowded with subjects who made one or two compromise judgments. But it was not. In every frame, 85% of subjects who chose an intermediate alternative made 3 to 15 compromise judgments (*SI Appendix*, Fig. S1), 8.8 on average (SD 4.7). When intermediate alternatives were available, compromisers chose them 58% of the time.

The prevalence of compromise judgments rules out the nonnegotiability hypothesis. It is evidence for a system that can resolve dilemmas with compromises (feature 1).

### Subjects Responded to Incentives.

**Fig. 3.**
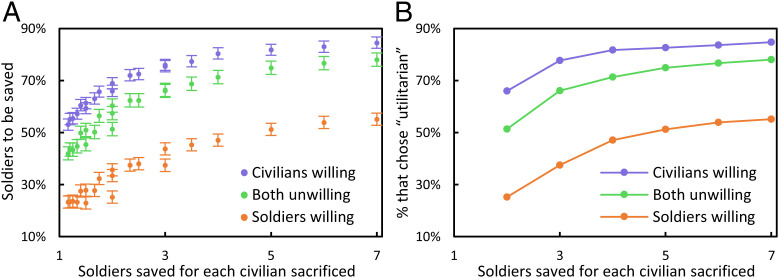
Subjects responded to the human cost of saving lives; the *x* axes represent *S*/*C*. (*A*) Percent of soldiers saved in each of 21 scenarios, with 95% CIs. As *S* varied, approximately the same proportion of soldiers were saved for each value of *S*/*C*. (*B*) All subjects responded to six scenarios in which they were forced to choose one of two extreme options; percent “utilitarian” responses for those scenarios.

Prediction 2 and conjecture 1 were also confirmed: The percent of soldiers that subjects felt should be saved increased with *S*/*C*. The effect is shown in Fig. 3*A*.

Six scenarios forced a choice between a deontic and a utilitarian solution, as in the standard design for moral dilemma experiments. When *S*/*C* increased, the percent of subjects choosing the utilitarian alternative increased, showing that incentives matter even when subjects are forced to make an extreme judgment, as shown in Fig. 3*B*.

These results show that the cognitive system in charge of moral judgment can respond to incentives (feature 2).

### Subjects Responded Coherently to Willingness.

Moral coherence is visible in the subjects’ average responses. Fig. 3*A* shows, for each scenario, the average percent of soldiers that subjects felt it was right to save in CW, BU, and SW. For every scenario, the percent to be saved was highest when civilians were willing, intermediate when both were unwilling, and lowest when soldiers were willing. The proportion of extreme responders reveals the same response pattern: “Utilitarians” were most common in CW and least common in SW; the reverse was true for “deontics” (Fig. 2*A*). These results support prediction 3 and conjecture 2.

### Willingness Changes the Subjective Value of Lives.

What weights did subjects assign to civilian and soldiers’ lives, and how did those weights change when willingness changed? To find out, we fitted the rightness function of a “representative agent”: one whose response to each scenario is the average of the subjects’ responses.

Fig. 3*A* depicts the representative agent’s 63 answers (21 per frame). Its rightness function matched a constant elasticity of substitution utility function (*SI Appendix* explains the estimation procedure). The function can be written as follows:

$$v(x,\beta )=\alpha^{\frac{1}{\sigma }}c^{\frac{\sigma -1}{\sigma }}+{\left(1-\alpha \right)}^{\frac{1}{\sigma }}s^{\frac{\sigma -1}{\sigma }}$$

where x=(c,s) is a solution, *c* and *s* are the numbers of surviving civilians and soldiers, and β=(α,σ) is a vector of parameters. The value of both parameters can differ across the three frames, because frames differ in two morally relevant variables: the willingness of civilians and the willingness of soldiers. The estimated values of *α* and *σ* give us a central tendency of subjects’ moral preferences; they are shown in Table 2.

**Table 2.** Parameters of representative rightness function

Condition	*α*	99% CI	*σ*	99% CI
Civilians willing	0.49	[0.47, 0.51]	1.98	[1.88, 2.08]
Both unwilling	0.61	[0.59, 0.63]	1.99	[1.89, 2.08]
Soldiers willing	0.80	[0.78, 0.81]	1.88	[1.78, 1.97]

Results from a nonlinear least squares regression. For details, see the *SI Appendix*.

Parameter α∈[0,1] represents the weight of civilians in moral value, whereas 1−α represents the weight of soldiers. If α=0.5, the agent cares equally about both types of people; if α>0.5, it cares more about civilians than soldiers. As expected, it valued civilians most highly when soldiers were willing, at an intermediate level—but still more highly than soldiers—when both were unwilling, and equally when civilians were willing.

Parameter σ≥0 is the elasticity of substitution: It regulates the sensitivity of the agent’s responses to changes in *S*/*C*. The elasticity of substitution did not vary with willingness. The high value of *σ* (approximately two) means that the representative agent is highly sensitive to incentives.

### Coherence of Individual Subjects.

We counted coherence violations for each individual as follows: Let $\left(c_{ij},s_{ij}\right)$ be the solution chosen by the subject in scenario *j* of frame *i*, where j=1,…,21 and i∈{BU,CW,SW}. A subject violated coherence in scenario *j* if $c_{\mathrm{cwj}}>c_{\mathrm{buj}}$ (a choice that entails the death of more unwilling than willing civilians) or if $s_{\mathrm{swj}}>s_{\mathrm{buj}}$ (a choice that entails the death of more unwilling than willing soldiers). By definition, each subject could violate coherence up to 21 times.

We could ask whether subjects’ responses were more coherent than expected by chance. This benchmark is too lax, however, because a subject that responds to incentives but does not maximize rightness would violate coherence less often than expected by chance alone. So we created a tougher benchmark: the judgments of simulated agents that respond to incentives exactly like subjects did on average. We call this benchmark “incentives + chance.” The 21 responses of a simulated agent were generated by independently resampling subjects’ choices for each separate scenario in a given frame. One million agents were created for BU and CW, and 1 million for BU and SW.

Subjects outperformed the incentives + chance benchmark by a wide margin (*SI Appendix*, Fig. S2); 69% of subjects never violated coherence, compared to less than 1% of simulated agents.

The 367 subjects who always made the same extreme judgment in both frames cannot violate coherence. The other 1,378 subjects could. Coherence was high for these subjects: 62% made zero violations, and 81% made coherent judgments in 90 to 100% of the 21 scenarios. Responding to incentives does not, by itself, produce these high levels of moral coherence: Only 20% of the incentives + chance agents were coherent in 90 to 100% of scenarios. See *SI Appendix*, Fig. S2 for subjects in CW/BU and SW/BU separately.

The average and individual-level results converge. Seventy-eight percent of subjects made different judgments in their two frames, confirming prediction 3. The cognitive system producing judgments responded to a morally relevant variable (feature 3), and did so coherently, confirming conjecture 2.

### Measuring Moral Rationality.

Judgments made by maximizing a well-behaved rightness function will respect GARP—the generalized axiom of revealed preferences (26, 27).^‡^ (See *SI Appendix* for a detailed explanation.) This mathematical fact allows for a crisp test of moral rationality.

We used the number of GARP violations made by each subject as a measure of irrationality. A subject makes a GARP violation by revealing an inconsistency of the form “**x** feels more right than **y**” and “**y** feels at least as right as **x**.” Revelations can be direct or indirect. Direct revelations involve two or more solutions that are in the same feasible set. Indirect revelations are inferred from transitivity chains that link three or more options that are not all in the same feasible set.

**Fig. 4.**
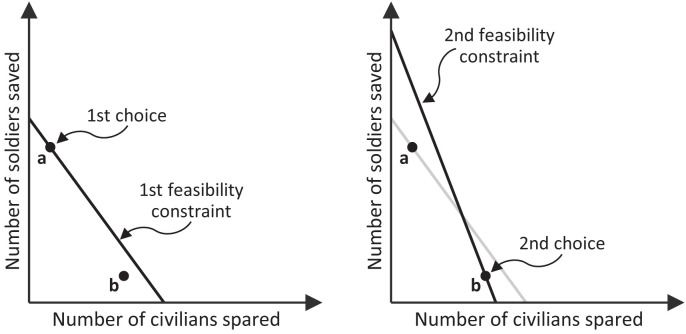
A simple type of GARP violation can occur when a subject faces two consecutive scenarios with intersecting feasibility constraints. Suppose the subject chooses **a** from the first feasibility constraint. This choice reveals that **a** feels more right than every solution below the constraint, including **b**. We can infer this even though **a** and **b** are not in the same feasible set (i.e., **b** was not available to be chosen), and **a** is not Pareto superior to **b**. This inference follows from the well-behavedness assumption (the proof is in *SI Appendix*). Next, the subject chooses **b** from the second feasibility constraint. This choice reveals that **b** feels more right than all the solutions below that constraint, including **a**. We can therefore infer that **b** feels more right than **a**, even though **a** is not feasible in the second scenario. These two inferences are contradictory: They imply that **a** feels both more right and less right than **b**. This inconsistency is a GARP violation.

Fig. 4 illustrates a simple type of GARP violation involving two consecutive scenarios. Respecting GARP becomes increasingly difficult as an agent faces more scenarios, due to the indirect revelation mechanism operating within and across them. Simulations show that, for the 21 scenarios, a subject can make up to 152 violations.

A perfectly functioning MTS will produce zero GARP violations. If it resides in a subject who occasionally makes a trembling hand mistake, then her judgments may fail to respect GARP fully. The fewer the GARP violations, the more evidence that a subject’s judgments were made by maximizing a well-behaved rightness function.

Making few GARP violations cannot be accomplished by chance: Random responders make a median of 61 violations. Responding to incentives is also insufficient to appear rational: Incentives + chance agents make a median of 45 violations.

### Subjects Made Rational Judgments.

Subjects outperformed both benchmarks, exhibiting high levels of rationality. The percent of subjects who never violated GARP was 71% in BU, 77% in CW, and 78% in SW. The corresponding figures were 0%, 1%, and 0% for the incentives + chance agents, and 1% for random responders. Moreover, 88% of subjects made no violations in at least one of their two frames. In each frame, 94% of subjects made seven or fewer violations, whereas <10% of incentives + chance agents did.

For detecting a cognitive system that maximizes rightness, zero violations is too strict a standard. The hypothesized cognitive system resides in a human, whose hand could tremble. So we compared subjects to simulated agents that are perfectly rational but make a single, random mistake. This *rational + 1 tremble* benchmark was created by resampling the subset of subjects with zero GARP violations in a given frame. In each iteration, we randomly changed one of the 21 choices of the drawn subject, to create one mistake per rational agent. We created 2 million agents for BU, 1 million for CW, and 1 million for SW.

For rational + 1 tremble agents, the median number of GARP violations was low: one in BU, two in CW, and one in SW. About 38% remained perfectly consistent, and ~77% made seven or fewer violations. Yet subjects scored better on all of these measures, as Table 3 and *SI Appendix*, Tables S2 and S3 show.

**Table 3.** Rationality in the BU frame

	Never	Made seven or	Median no.	90th	95th
Individuals	violated GARP, %	fewer violations, %	of violations	percentile	percentile
All subjects	71	94	0	5	10
Compromisers	59	92	0	7	15
Flip-floppers	53	88	0	9	17
*Benchmarks*					
Random responders	~0	1	61	90	97
Incentives + chance	~0	5	45	78	86
Rational + 1 tremble	39	76	1	15	23
Dual + compromises	39	62	3	26	35

### Compromisers Provide a Critical Test.

Subjects who respect GARP are expected on the MTS model. Is the same true of the dual-process model?

Extreme responders (who always respect GARP) can be explained by both models. The dual-process model will produce a deontic response profile if an alarm bell emotion preempts reasoning every time, and a utilitarian profile if reasoning always prevails. An MTS can also produce these profiles. A rightness function that assigns zero weight to soldiers [v(c,s)=c] will produce a deontic profile; one that assigns zero weight to civilians [v(c,s)=s] will produce a utilitarian profile.^§^

Both models can also explain GARP-respecting flip-floppers, but only if we make the charitable assumption that a dual process somehow responds to incentives. The dual-process model will produce them if and only if emotion trumps reason when *S*/*C* is below a threshold. An MTS will produce them if it constructs a linear rightness function, with positive weights assigned to both soldiers and civilians [v(c,s)=αc+(1−α)s, where 0<α<1]. When *S*/*C* exceeds a threshold, the optimal solution is utilitarian; when *S*/*C* is below that threshold, the optimal solution is deontic (see *SI Appendix* for the proof).

Most subjects were compromisers, however. Their rationality—or lack thereof—provides a critical test between the MTS hypothesis and the dual-process model.

The MTS hypothesis predicts that compromisers will be rational: They will make few, if any, GARP violations.^¶^ The dual-process model does not predict that compromisers will exist, let alone be rational. It can attribute their existence to hand trembles, of course. But compromises made by mistake create many GARP violations, as we show below.

### Compromisers Made Rational Judgments.

Most compromisers never violated GARP: 59% in BU, 64% in CW, and 58% in SW. In every condition, ~90% of compromisers made seven or fewer violations. By contrast, ~0% of incentives + chance agents made zero violations, and less than 10% made seven or fewer violations.

The compromisers even outperformed the rational + 1 tremble agents. In all frames, the percent of compromisers with zero violations was higher by at least 20 points. The percent of compromisers who made seven or fewer violations was also higher: ~90% of them compared to 77% of rational + 1 tremble agents. See Table 3 and *SI Appendix*, Tables S2 and S3.

Of the 1,237 compromisers, 85% made zero violations in at least one frame; 81%, if we exclude cases in which the same extreme option was chosen 21 times (49). Even if their hand trembled in one condition, their flawlessly rational performance in the other evidences a cognitive system that maximizes rightness.

The rationality of compromisers confirms prediction 4.

**Fig. 5.**
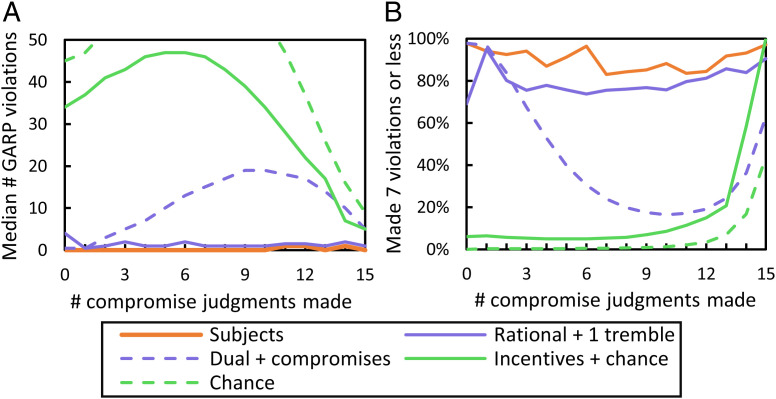
Subjects made rational responses, no matter how many compromise judgments they made (BU). (*A*) The median number of GARP violations as a function of number of compromises made; (*B*) percent making few violations (seven or fewer). Regardless of difficulty (the curve for chance), the median number of GARP violations by compromisers was approximately zero, and the percent making few violations was high.

A fine-grained analysis of performance as a function of difficulty supports the optimization hypothesis even more strongly. How difficult it is to respect GARP varies with the number of compromises an agent makes. The median number of violations made by random responders is an indicator of difficulty. As Fig. 5*A* shows, difficulty depends on the number of compromises made. It is high for all numbers of compromises, and has an inverted U shape.

Difficulty thus defined should not matter for an MTS. When the input to MAX is a well-behaved rightness function, its optimization algorithm will identify the most right solution in a scenario. That process does not depend on how many compromises MAX produced in previous scenarios. Respecting GARP is a cost-free byproduct of optimization. Effortful, conscious reasoning is unnecessary to remain consistent across scenarios.

Difficulty would matter, however, if compromise judgments were produced by nonoptimizing algorithms: heuristics, inflexible emotions, or deliberative reasoning. Respecting GARP is not a byproduct of their design, so avoiding violations would require a backward-looking algorithm. Each new judgment would have to be made factoring in all the previous ones. It follows that GARP violations should increase with difficulty. A heuristic that responds to incentives without looking backward is a case in point: GARP violations increased with difficulty for the incentives + chance agents.

As Fig. 5*A* and *SI Appendix*, Fig. S3 show, compromisers made highly rational judgments regardless of the number of compromises they made: Their median number of violations was zero in 87% of cases, and two at most. Remarkably, compromisers outperformed the rational + 1 tremble agents, the most exacting benchmark. Respecting GARP across all levels of difficulty is a signature of optimization.

### A Dual Process Cannot Create Rational Compromisers.

The dual-process model does not specify how the two processes would respond to incentives, so it makes no specific predictions about GARP violations. We can, however, bracket the violations expected between two bounds. Random flip-flopping sets an upper bound: Since *S*/*C* and *S* vary unpredictably across scenarios, an alarm bell could go off unpredictably in response. This would create a median of 45 violations, not the median of 0 found for subjects. A lower bound is set by extreme judgments that are rational, with compromises as hand trembles.

To quantify this lower bound, we created a *dual + k compromises* benchmark: simulated agents created by resampling the extreme responders and GARP-respecting flip-floppers in a given frame. For each agent, we randomly created *k* compromises (range: 0 to 15). For comparability, we made the distribution of compromises for the bootstrapped samples the same as for subjects. These distributions are depicted in Fig. 2*B* and *SI Appendix*, Fig. S1. We created 2 million agents for BU, 1 million for CW, and 1 million for SW.

The performance profile for dual + *k* compromise agents has an inverted U shape: They make more violations when the task is more difficult (Fig. 5*A* and *SI Appendix*, Fig. S3). This shape is the fingerprint of systems that do not optimize. It contrasts with the performance profile for subjects as a function of *k*, which is flat at zero violations.

We also examined the percent of subjects and dual + *k* compromise agents who made seven or fewer violations as a function of *k*. The results are presented in Fig. 5*B* and *SI Appendix*, Fig. S3. At the highest level of difficulty (*k* = 6, 7; median = 63 violations), over 85% of subjects made seven or fewer violations (BU: 90%; CW: 92%; SW: 86%), whereas a minority of dual + *k* compromise agents did (BU: 27%; CW:21%; SW: 37%). Subjects were far more rational than expected from a dual process + mistakes model.

The prevalence of rational compromisers—ones who respected GARP even when it was most difficult to do so—is strong evidence of a cognitive system that weighs different moral values and chooses a most right solution (feature 4).

### Moral Judgments Were Intuitive.

Deliberative reasoning cannot explain GARP-respecting compromisers. It would require constructing and maintaining a consistent preference order that expands as each new judgment is made. In this experiment, your working memory would have to hold a growing record of up to 435 revealed preference relations. This is because the 21 scenarios include 29 different solutions, and each solution can be in a preference relationship with itself and all other solutions. To avoid violating GARP, you would have to check each feasible solution in a scenario for possible violations, choose one that is consistent with all previously revealed preferences, and then update the record accordingly. The cognitive load of this task makes it intractable. The only way to ensure no GARP violations through deliberative reasoning is to make the same extreme judgment 21 times.^#^

An intuitive optimization process can produce rational compromisers; System 2 reasoning cannot.

### Rightness Functions Are Temporary Representations.

Judgments that respect GARP in both frames, yet vary across frames, are evidence that rightness functions are constructed on the fly. To test this, we examined the 78% of subjects whose judgments varied with willingness. These subjects were rational within each frame: Most never violated GARP (BU: 63%; CW: 72%; SW: 71%), and 92% made seven or fewer violations. Indeed, most of them were rational in both frames (zero violations: 49%; seven or fewer: 86%), confirming prediction 5. This indicates that their MTS had constructed, and maximized, different rightness functions in each willingness frame. The initial function persisted while subjects made (rational) judgments in the first frame they faced, but it was replaced by a different function when they faced the second frame.

## Conclusion

The results of the war dilemma revealed a previously unknown cognitive competence: a moral tradeoff system. It is composed of three subsystems: MV, FS, and MAX. MV integrates conflicting values into a rightness function: a temporary representation that maps each conceivable solution onto a level of rightness. In parallel, FS constructs a feasible set: a temporary representation of the subset of conceivable solutions that are perceived as available. MAX uses these representations to identify a feasible solution with the maximum level of rightness, delivering it as an intuitive judgment.

How do we know that the MTS works like this?

The results support every prediction this model makes, including unique ones. But the most decisive evidence is that the vast majority of subjects were rational in the sense of GARP: They made few GARP violations. They were rational no matter what mix of extreme and compromise judgments they made, and remained rational across frames—even when their judgments changed with the willingness of soldiers or civilians. This is the signature of a process that maximizes rightness.

We considered five alternative hypotheses; none could explain the results—especially rational judgments by compromisers. Responding randomly produces many GARP violations; so does responding to incentives without maximizing rightness. Three inflexible rules—always choose deontic, always choose utilitarian, and flip-flop at a threshold—can produce extreme judgments that respect GARP. But no inflexible rule can produce GARP-respecting compromises. This includes the inflexible command, do not harm, issued by System 1 of Greene’s dual-process model (14). Yet more than 70% of subjects made compromises, and when they did, over 90% made rational judgments (zero to seven violations; the maximum possible number is 152).

The dual-process model proposed by Greene and colleagues (14, 15) makes several unique predictions, all contradicted by the data.

A straightforward version of their model predicts that the war dilemma will always elicit extreme judgments, because its conflicting values cannot be “negotiated by weighing preferences against each other” (15). This rules out compromise judgments, because they result from weighing moral preferences: Compromises strike a balance between conflicting values by partially satisfying both. It follows that compromise judgments will be infrequent and unsystematic, because they are noise. The fact that compromises were both frequent and rational contradicts these predictions. Indeed, compromise judgments that respect GARP are evidence of a rightness function that assigns positive weights to conflicting values (in this case, the lives of civilians and soldiers). The MTS “negotiates” the conflicting values by assigning a rightness level to each conceivable solution, including the intermediate ones.

A version of the dual-process model that jettisons the nonnegotiability hypothesis is refuted as well. In that model, flexibility—the ability to respond to context by integrating multiple considerations—requires conscious, deliberative reasoning. This implies that only System 2 could produce compromise judgments. But this version of the model also fails to explain the prevalence of rational compromisers. If intermediate solutions are chosen, deliberative reasoning cannot prevent GARP violations across 21 scenarios with intersecting feasibility constraints (49). The cognitive load of the task makes it impossible, not only for Greene’s model, but for any model that seeks to attribute the results to deliberative reasoning.

An original feature of the MTS hypothesis is that rightness functions and feasible sets are temporary mental representations, constructed on the fly for a specific dilemma. We know that the MTS can construct new rightness functions on the fly because most subjects changed their judgments when the willingness frame was different but the scenarios were the same. We know that the MTS can construct new feasible sets on the fly because, within a frame, most subjects responded to different scenarios by changing their judgments. This flexibility necessitates three subsystems: two subsystems that construct the temporary representations, and a third subsystem that uses them to identify an optimal solution.

These findings open many questions. How does MV integrate conflicting values? Does it have a library of functional forms to draw on, with free parameters calculated on the fly (50)? Does FS represent the available options as a set, or in summary form—analogous to the “budget lines” in Fig. 1*D*?

Finally, the three-subsystem architecture proposed here may provide a useful template for psychologists and social scientists studying choice in domains outside moral psychology. An architecture like this could be present in many other cognitive systems, each specialized for a different domain of choice.

## Materials and Methods

Study procedures were approved by the University of California, Santa Barbara Institutional Review Board. Participants gave fully informed consent before answering the survey. The survey was implemented in Qualtrics. US adults were recruited through Amazon MTurk in 2016. Subjects were paid $1. A session lasted ~15 min. Criteria for inclusion were set in advance. The dataset for analysis was all subjects who completed the survey and correctly answered two attention checks and an English language comprehension question. N=1,745 met these criteria: 54% female; mean age = 36 y (SD 12 y), range 18 y to 87 y. Subjects were randomly assigned to the different treatments. See *SI Appendix* for full text of the instrument. Data are available at Open Science Framework (OSF; https://osf.io/kd34j/).

## Supplementary Material

Supplementary File

## Data Availability

Excel spreadsheet and codes have been deposited in OSF (https://osf.io/kd34j/) (51).

## Footnotes

^*^Extreme vs. compromise judgments would be called corner vs. interior solutions in rational choice theory. Later, we will refer to maximizing a “rightness function” rather than a utility function, because “utility” is too easily confused with utilitarian in the moral sense. Maximizing a rightness function can result in a compromise or deontic judgment—it does not imply one is maximizing a “utilitarian” moral value, such as saving the most lives.

^†^Because a rightness function ranks all conceivable solutions to a dilemma, many different scenarios—each representing different harms and benefits to the parties involved—can be evaluated in advance of discovering which one pertains when a decision must be made. This feature enables prospective thinking, as well as on-the-spot judgments.

^‡^Well behavedness is an auxiliary assumption. It consists of three mathematical properties: continuity, nonsatiation, and convex indifference curves. This assumption biases the test against the MTS hypothesis: Judgments produced by maximizing a function with different properties may satisfy the axioms of rational choice (48), yet violate GARP.

^§^Assigning zero weight is only one of many ways the MTS can produce an extreme profile.

^¶^An MTS can produce compromises by maximizing a rightness function with strictly convex indifference curves, for example; see *SI Appendix*.

^#^Economists participating in GARP studies know this. Harbaugh et al. (49) could not use economists as a control group, because many were more concerned with appearing rational than with choosing the options that they preferred. They avoided intermediate options entirely, confessing afterward that they chose the same extreme option every time to prevent “embarrassing” GARP violations.
