# Supplementary material for: A moral trade-off system produces intuitive judgments that are rational and coherent and strike a balance between conflicting moral values
Source: Proc Natl Acad Sci U S A. 2022 Oct 10;119(42):e2214005119. doi: 10.1073/pnas.2214005119 (PMC9586309; doi:10.1073/pnas.2214005119)
Supplement: Supplementary File [file pnas.2214005119.sapp.pdf]

Supplementary information for

A moral tradeoff system produces intuitive judgments that are rational, coherent, and strike a balance between conflicting moral values

Ricardo Andrés Guzmán[a,*], María Teresa Barbato[a], Daniel Sznycer[b], and Leda Cosmides[c,*]

[a]Centro de Investigación en Complejidad Social, Universidad del Desarrollo, Santiago.
[b]Department of Psychology, University of Montreal and Oklahoma Center for Evolutionary Analysis, Department of Psychology, Oklahoma State University, Stillwater, OK 74078-3064.
[c]Center for Evolutionary Psychology, University of California, Santa Barbara.
[*]Corresponding authors. E-mail: rguzman@udd.cl, cosmides@ucsb.edu.

September 2, 2022

Contents

1 Additional figures and tables **2**

2 Written description of the war dilemma **6**

2.1 Both unwilling followed by civilians willing 6
2.2 Both unwilling followed by soldiers willing 7
2.3 Civilians willing followed by both unwilling 8
2.4 Soldiers willing followed by both unwilling 9

3 Scenarios of the war dilemma **11**

4 Moral rationality in the war dilemma **13**

4.1 Rational choice theory in a nutshell 13
4.2 *Homo economicus* 14
4.3 What are preferences? 14
4.4 Moral preferences 15
4.5 Properties of a preference order 16
4.6 Indifference and strict preference 16
4.7 Utility functions 17
4.8 Rightness functions are utility functions 18
4.9 Testing for consistency requires auxiliary assumptions 19
4.10 Well-behavedness 19
4.11 Utilitarian and balanced rightness functions 21
4.12 Feasible solutions to the war dilemma 21
4.13 Compromises judgments as rightness maximizing choices 22
4.14 Deontic moral values 23
4.15 Rational moral flip-flopping 23
4.16 Revealed preferences, hand trembles, and inconsistency 24
4.17 Preference inference rules 25
4.18 The generalized axiom of revealed preferences 26
4.19 Discretized solutions 31
4.20 Counting GARP violations 31

5 The representative agent's rightness function **33**

1 Additional figures and tables

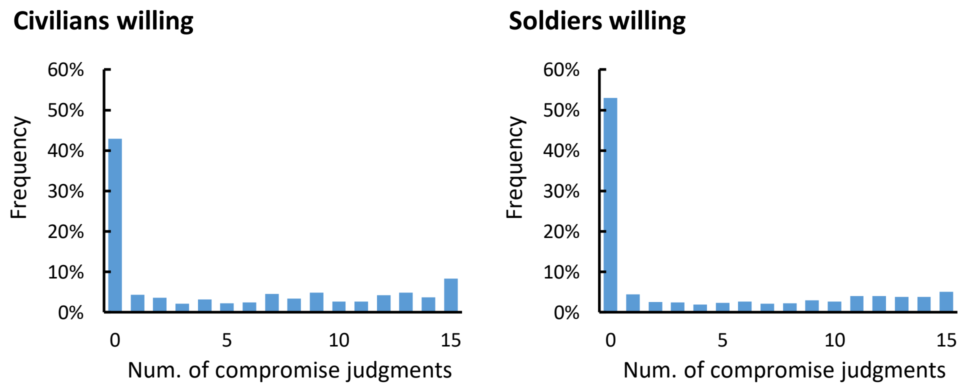

Figure S1. Frequency of compromise judgments made in the civilians willing and soldiers willing conditions.

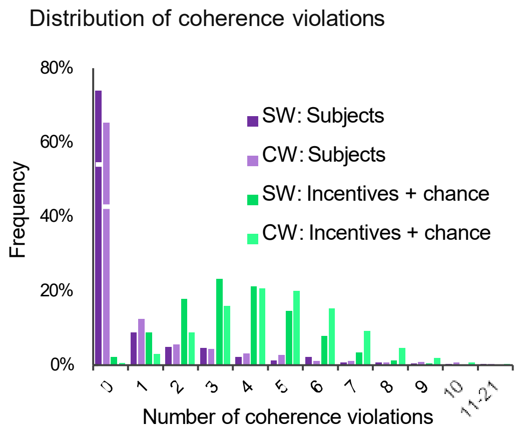

Figure S2. Number of coherence violations made by subjects and simulated agents. Most subjects responded coherently to changes in *willingness*. (Sections above the white segments represent subjects who always made the same extreme judgment in both conditions.

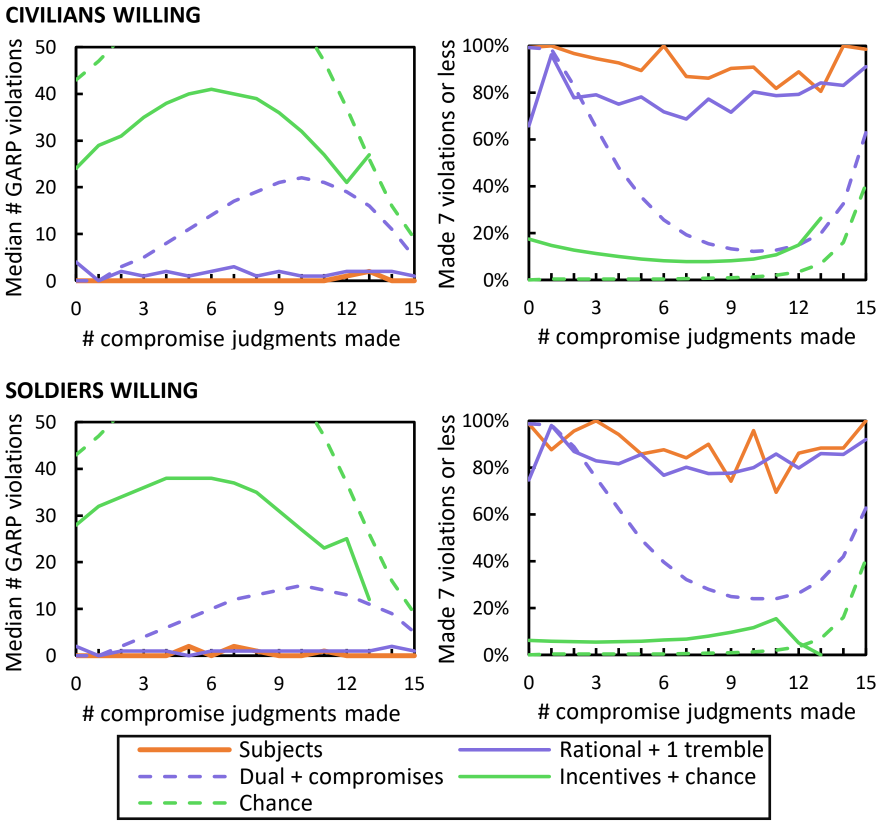

Figure S3. Performance of subjects and simulated agents in the war dilemma.

Table S1. Scenarios of the war dilemma

Scenario #	Soldiers at risk (S)	Soldiers saved for each civilian sacrificed (S / C)	Alternatives (civil. sacrificed, sold. dead)	Feasible set (civil. spared, sold. saved)	Pareto inferior solutions (civil. spared, sold. saved)
1	2	2.00	(0, 2) (1, 0)	(1, 0) (0, 2)	(0, 0) (0, 1)
2	3	3.00	(0, 3) (1, 0)	(1, 0) (0, 3)	(0, 0) (0, 1) (0, 2)
3	3	1.50	(0, 3) (1, 2) (2, 0)	(2, 0) (1, 1) (0, 3)	(0, 0) (1, 0) (0, 1) (0, 2)
4	4	4.00	(0, 4) (1, 0)	(1, 0) (0, 4)	(0, 0) (0, 1) (0, 2) (0, 3)
5	4	2.00	(0, 4) (1, 2) (2, 0)	(2, 0) (1, 2) (0, 4)	(0, 0) (1, 0) (0, 1) (1, 1) (0, 2) (0, 3)
6	4	1.33	(0, 4) (1, 3) (2, 2) (3, 0)	(3, 0) (2, 1) (1, 2) (0, 4)	(0, 0) (1, 0) (2, 0) (0, 1) (1, 1) (0, 2) (0, 3)
7	5	5.00	(0, 5) (1, 0)	(1, 0) (0, 5)	(0, 0) (0, 1) (0, 2) (0, 3) (0, 4)
8	5	2.50	(0, 5) (1, 3) (2, 0)	(2, 0) (1, 2) (0, 5)	(0, 0) (1, 0) (0, 1) (1, 1) (0, 2) (0, 3) (0, 4)
9	5	1.67	(0, 5) (1, 4) (2, 2) (3, 0)	(3, 0) (2, 1) (1, 3) (0, 5)	(0, 0) (1, 0) (2, 0) (0, 1) (1, 1) (0, 2) (1, 2) (0, 3) (0, 4)
10	5	1.25	(0, 5) (1, 4) (2, 3) (3, 2) (4, 0)	(4, 0) (3, 1) (2, 2) (1, 3) (0, 5)	(0, 0) (1, 0) (2, 0) (3, 0) (0, 1) (1, 1) (2, 1) (0, 2) (1, 2) (0, 3) (0, 4)
11	6	6.00	(0, 6) (1, 0)	(1, 0) (0, 6)	(0, 0) (0, 1) (0, 2) (0, 3) (0, 4) (0, 5)
12	6	3.00	(0, 6) (1, 3) (2,0)	(2, 0) (1, 3) (0, 6)	(0, 0) (1, 0) (0, 1) (1, 1) (0, 2) (1, 2) (0, 3) (0, 4) (0, 5)
13	6	2.00	(0, 6) (1, 4) (2,2) (3,0)	(3, 0) (2, 2) (1, 4) (0, 6)	(0, 0) (1, 0) (2, 0) (0, 1) (1, 1) (2, 1) (0, 2) (1, 2) (0, 3) (1, 3) (0, 4) (0, 5)
14	6	1.50	(0, 6) (1, 5) (2,3) (3,2) (4,0)	(4, 0) (3, 1) (2, 3) (1, 4) (0, 6)	(0, 0) (1, 0) (2, 0) (3, 0) (0, 1) (1, 1) (2, 1) (0, 2) (1, 2) (2, 2) (0, 3) (1, 3) (0, 4) (0, 5)
15	6	1.20	(0, 6) (1, 5) (2, 4) (3, 3) (4, 2) (5, 0)	(5, 0) (4, 1) (3, 2) (2, 3) (1, 4) (0, 6)	(0, 0) (1, 0) (2, 0) (3, 0) (4, 0) (0, 1) (1, 1) (2, 1) (3, 1) (0, 2) (1, 2) (2, 2) (0, 3) (1, 3) (0, 4) (0, 5)
16	7	7.00	(0, 7) (1, 0)	(1, 0) (0, 7)	(0, 0) (0, 1) (0, 2) (0, 3) (0, 4) (0, 5) (0, 6)
17	7	3.50	(0, 7) (1, 4) (2, 0)	(2, 0) (1, 3) (0, 7)	(0, 0) (1, 0) (0, 1) (1, 1) (0, 2) (1, 2) (0, 3) (0, 4) (0, 5) (0, 6)
18	7	2.33	(0, 7) (1, 5) (2, 3) (3, 0)	(3, 0) (2, 2) (1, 4) (0, 7)	(0, 0) (1, 0) (2, 0) (0, 1) (1, 1) (2, 1) (0, 2) (1, 2) (0, 3) (1, 3) (0, 4) (0, 5) (0, 6)
19	7	1.75	(0, 7) (1, 6) (2, 4) (3, 2) (4, 0)	(4, 0) (3, 1) (2, 3) (1, 5) (0, 7)	(0, 0) (1, 0) (2, 0) (3, 0) (0, 1) (1, 1) (2, 1) (0, 2) (1, 2) (2, 2) (0, 3) (1, 3) (0, 4) (1, 4) (0, 5) (0, 6)
20	7	1.40	(0, 7) (1, 6) (2, 5) (3, 3) (4, 2) (5, 0)	(5, 0) (4, 1) (3, 2) (2, 4) (1, 5) (0, 7)	(0, 0) (1, 0) (2, 0) (3, 0) (4, 0) (0, 1) (1, 1) (2, 1) (3, 1) (0, 2) (1, 2) (2, 2) (0, 3) (1, 3) (2, 3) (0, 4) (1, 4) (0, 5) (0, 6)
21	7	1.17	(0, 7) (1, 6) (2, 5) (3, 4) (4, 3) (5, 2) (6, 0)	(6, 0) (5, 1) (4, 2) (3, 3) (2, 4) (1, 5) (0, 7)	(0, 0) (1, 0) (2, 0) (3, 0) (4, 0) (5, 0) (0, 1) (1, 1) (2, 1) (3, 1) (4, 1) (0, 2) (1, 2) (2, 2) (3, 2) (0, 3) (1, 3) (2, 3) (0, 4) (1, 4) (0, 5) (0, 6)

Note: All quantities in millions of lives. In a scenario, each alternative presented to subjects corresponds to a solution in the feasible set. Each solution is an ordered pair (c, s), where c is the number of civilians spared and s is the number of soldiers saved. The corresponding alternative is the ordered pair ($C - c$, $S - s$), where $C - c$ is the number of civilians sacrificed and $S - s$ is the number of soldiers dead. The "Pareto inferior solutions" are inferior to *at least one* feasible solution. There are only 29 different solutions, and almost all of them appear in more than one scenario, either as a feasible solution or a Pareto inferior solution.

Table S2. Rationality in the civilians willing condition.

Individuals	Never violated GARP	Made 7 viols. or less	Median num. of violations	90th Percentile	95th Percentile
All subjects	78%	95%	0	3	7
Compromisers	64%	92%	0	6	15
Flip-floppers	67%	93%	0	6	15
Benchmarks					
Random responders	~0%	1%	61	90	97
Incentives + chance	1%	9%	38	74	82
Rational + 1 tremble	39%	73%	2	16	24
Dual + compromises	47%	65%	2	27	35

Table S3. Rationality in the soldiers willing condition

Individuals	Never violated GARP	Made 7 viols. or less	Median num. of violations	90th Percentile	95th Percentile
All subjects	77%	94%	0	3	8
Compromisers	58%	88%	0	8	14
Flip-floppers	54%	94%	0	6	13
Benchmarks					
Random responders	~0%	1%	61	90	97
Incentives + chance	~0%	6%	36	65	73
Rational + 1 tremble	36%	82%	1	11	16
Dual + compromises	54%	75%	0	19	27

2 Written description of the war dilemma

Here we will present the written description of the dilemma, as presented to subjects. Yellow highlight indicates phrases that varied between the three conditions and four treatments (a treatment consists of the baseline condition and one of two variants, shown to subjects in that order or in reverse order).

2.1 Both unwilling followed by civilians willing

Two foreign countries, A and B, have been at war for several years (you are not a citizen of either of these two countries). The war was initiated by the rulers of country B, against the will of the civilian population. The war has been bloody: Millions have died during the conflict, which so far had been deadlocked. Recently, the military equilibrium has broken, and it is now certain that Country A will win the war sooner or later. The question is how, when, and at what cost. Country A has two strategies available. Country A could use one, the other, or a combination of both. The first strategy is to attack the opposing army with conventional weapons, preventing civilian casualties almost completely. If Country A applies this strategy, the war will continue for some time (perhaps years). The delay in the end of the war will cause the deaths of a great number of soldiers of both sides. Of the soldiers that die, about half will be from country A and half from country B. Nearly all are young soldiers who were forced to join the army against their will, and are desperate to return to their families. The second strategy available to Country A is to bomb cities of Country B, killing civilians (who opposed the war from the beginning) and almost no soldiers. This strategy would demoralize Country B and force it to surrender quickly. The war would end soon. There is a third approach. Country A could bring the war to an end by using both strategies, resulting in the deaths of some civilians and some soldiers. The more civilians are sacrificed (killed) during the bombings, the sooner Country B will surrender, and the fewer soldiers will die on the battlefield. How should Country A end the war?

[Before answering the scenarios of the next condition(civilians willing), the subjects saw the following message.]

Now imagine a war in which everything is the same as before, except for the original attitude of the civilians of the aggressor country toward the war. The situation is as follows: The war was initiated by the rulers of country B, with the support

of the civilian population. As in the previous case, the armies of both countries are made up of young soldiers who were forced to join the army against their will, and are desperate to return to their families. Remember that Country A could bring the war to an end in three ways:

1. By attacking the opposing army with conventional weapons, in which case many soldiers would die, but no civilians would die.
2. By bombing cities, in which case many civilians would die, but no soldiers.
3. By a combination of both strategies, in which case some soldiers and some civilians would die.

Also remember that of the soldiers that die, about half will be from country A and half from country B. How should Country A end the war?

2.2 Both unwilling followed by soldiers willing

Two foreign countries, A and B, have been at war for several years (you are not a citizen of either of these two countries). The war was initiated by the rulers of country B, against the will of the civilian population. The war has been bloody: Millions have died during the conflict, which so far had been deadlocked. Recently, the military equilibrium has broken, and it is now certain that Country A will win the war sooner or later. The question is how, when, and at what cost. Country A has two strategies available. Country A could use one, the other, or a combination of both. The first strategy is to attack the opposing army with conventional weapons, preventing civilian casualties almost completely. If Country A applies this strategy, the war will continue for some time (perhaps years). The delay in the end of the war will cause the deaths of a great number of soldiers of both sides. Of the soldiers that die, about half will be from country A and half from country B. Nearly all are young soldiers who were forced to join the army against their will, and are desperate to return to their families. The second strategy available to Country A is to bomb cities of Country B, killing civilians (who opposed the war from the beginning) and almost no soldiers. This strategy would demoralize Country B and force it to surrender quickly. The war would end soon. There is a third approach. Country A could bring the war to an end by using both strategies, resulting in the deaths of some civilians and some soldiers. The more civilians are sacrificed (killed) during the bombings, the sooner Country B

will surrender, and the fewer soldiers will die on the battlefield. How should Country A end the war?

[Before answering the scenarios of the next condition (soldiers willing), the subjects saw the following message.]

Now imagine a war in which everything is the same as before, except for the motivation of the soldiers. The situation is as follows: The armies of both countries are made up of young soldiers who volunteered, and are willing to fight for their country. As in the previous case, the civilian population of country B, the aggressor, did not support the war. Remember that Country A could bring the war to an end in three ways:

1. By attacking the opposing army with conventional weapons, in which case many soldiers would die, but no civilians would die.

2. By bombing cities, in which case many civilians would die, but no soldiers.

3. By a combination of both strategies, in which case some soldiers and some civilians would die.

Also remember that of the soldiers that die, about half will be from country A and half from country B. How should Country A end the war?

2.3 Civilians willing followed by both unwilling

Two foreign countries, A and B, have been at war for several years (you are not a citizen of either of these two countries). The war was initiated by the rulers of country B, with the support of the civilian population. The war has been bloody: Millions have died during the conflict, which so far had been deadlocked. Recently, the military equilibrium has broken, and it is now certain that Country A will win the war sooner or later. The question is how, when, and at what cost. Country A has two strategies available. Country A could use one, the other, or a combination of both. The first strategy is to attack the opposing army with conventional weapons, preventing civilian casualties almost completely. If Country A applies this strategy, the war will continue for some time (perhaps years). The delay in the end of the war will cause the deaths of a great number of soldiers of both sides. Of the soldiers that die, about half will be from country A and half from country B. Nearly all are young soldiers who were forced to join the army against their will, and are desperate

to return to their families. The second strategy available to Country A is to bomb cities of Country B, killing civilians (who supported the war from the beginning) and almost no soldiers. This strategy would demoralize Country B and force it to surrender quickly. The war would end soon. There is a third approach. Country A could bring the war to an end by using both strategies, resulting in the deaths of some civilians and some soldiers. The more civilians are sacrificed (killed) during the bombings, the sooner Country B will surrender, and the fewer soldiers will die on the battlefield. How should Country A end the war?

[Before answering the scenarios of the next condition (both unwilling), the subjects saw the following message.]

Now imagine a war in which everything is the same as before, except for the original attitude of the civilians of Country B (the aggressor) toward the war. The situation is as follows: The war was initiated by the rulers of country B, against the will of the civilian population. As in the previous case, the armies of both countries are made up of young soldiers who were forced to join the army against their will, and are desperate to return to their families. Remember that Country A could bring the war to an end in three ways:

1. By attacking the opposing army with conventional weapons, in which case many soldiers would die, but no civilians would die.

2. By bombing cities, in which case many civilians would die, but no soldiers.

3. By a combination of both strategies, in which case some soldiers and some civilians would die.

Also remember that of the soldiers that die, about half will be from country A and half from country B. How should Country A end the war?

2.4 Soldiers willing followed by both unwilling

Two foreign countries, A and B, have been at war for several years (you are not a citizen of either of these two countries). The war was initiated by the rulers of country B, against the will of the civilian population. The war has been bloody: Millions have died during the conflict, which so far had been deadlocked. Recently, the military equilibrium has broken, and it is now certain that Country A will win the war sooner or later. The question is how, when, and at what cost. Country A has two strategies

available. Country A could use one, the other, or a combination of both. The first strategy is to attack the opposing army with conventional weapons, preventing civilian casualties almost completely. If Country A applies this strategy, the war will continue for some time (perhaps years). The delay in the end of the war will cause the deaths of a great number of soldiers of both sides. Of the soldiers that die, about half will be from country A and half from country B. Nearly all are young soldiers who volunteered, and are willing to fight for their country. The second strategy available to Country A is to bomb cities of Country B, killing civilians (who opposed the war from the beginning) and almost no soldiers. This strategy would demoralize Country B and force it to surrender quickly. The war would end soon. There is a third approach. Country A could bring the war to an end by using both strategies, resulting in the deaths of some civilians and some soldiers. The more civilians are sacrificed (killed) during the bombings, the sooner Country B will surrender, and the fewer soldiers will die on the battlefield. How should Country A end the war?

[Before answering the scenarios of the next condition (both unwilling), the subjects saw the following message.]

Now imagine a war in which everything is the same as before, except for the motivation of the soldiers. The situation is as follows: The armies of both countries are made up of young soldiers who were forced to join the army against their will, and are desperate to return to their families. As in the previous case, the civilian population of country B, the aggressor, did not support the war. Remember that Country A could bring the war to an end in three ways:

1. By attacking the opposing army with conventional weapons, in which case many soldiers would die, but no civilians would die.

2. By bombing cities, in which case many civilians would die, but no soldiers.

3. By a combination of both strategies, in which case some soldiers and some civilians would die.

Also remember that of the soldiers that die, about half will be from country A and half from country B. How should Country A end the war?

3 Scenarios of the war dilemma

In each condition, subjects were given 21 scenarios of the war dilemma, summarized in table S1. A scenario is a multiple-choice question that offers a minimum of two and a maximum of seven alternatives for ending a war. An alternative consists of x civilians sacrificed and y soldiers killed. The number of civilians that could be sacrificed ranges from zero to C, and the number of soldiers that could be killed ranges from S to zero, where $S > C$. The values of C and S vary from scenario to scenario.

The more civilians are sacrificed in a given scenario, the fewer soldiers will die. Each civilian death saves $S/C > 1$ soldiers, approximately. It follows that total deaths are minimized when C civilians are sacrificed (the maximum possible number). Parameters S and S/C specify the incentives of the dilemma.[1] Saving one soldier requires sacrificing C/S civilians; S/C is the reciprocal of the relative price of saving a soldier—it expresses how many soldiers will be saved by sacrificing a single civilian.[2] C, on the other hand, is analogous to what economists would call "income": It is the number of civilian lives that are available to exchange for soldiers' lives.

Figure S4 shows the description of a scenario presented to subjects. Total deaths in this scenario range from $C = 4$ million (all civilians) to $S = 6$ million (all soldiers).

Alternatives were presented to subjects expressed in terms of bads (human deaths). To analyze subjects' responses using a revealed preference method, we re-expressed the alternatives as their corresponding goods (human lives).

We use the term "solution" to denote an outcome of the war expressed in terms of lives. Solution (c, s) is a bundle of c civilian lives and s soldiers' lives.[3] If an alternative is "x civilians sacrificed and y soldiers killed," its corresponding feasible solution is $c = C - x$ civilians spared and $s = S - y$ soldiers saved. For example, the fourth alternative in Fig. S4—3 million sacrificed civilians and 2 million dead soldiers—corresponds to solution $(1, 4)$; that is, 1 million civilians spared and 4 million soldiers saved.

[1] In economics, the incentives would be the relative price of two goods and the income available to spend on them.

[2] The reciprocal of price is called "cost-effectiveness" in economics. The greater S/C is, the more lives are saved by sacrificing one civilian.

[3] "Bundle" is a term of art in microeconomics. A bundle is an n-dimensional vector or n-tuple containing non-negative quantities of n goods.

1. If no civilians are sacrificed, 6 million soldiers will die on the battlefield.
2. To end the war and save all the soldiers, 4 million civilians would have to be sacrificed.
3. For every 4 civilians sacrificed during the bombings, 6 soldiers less will die on the battlefield, approximately.

Remember that half of the soldiers who die are from each country, and that you are not a citizen of either country.

Given the above scenario, choose the combination of dead soldiers and sacrificed civilians that feels morally right to you.

- ○ 6 million soldiers / 0 civilians
- ○ 5 million soldiers / 1 million civilians
- ○ 3 million soldiers / 2 million civilians
- ○ 2 million soldiers / 3 million civilians
- ○ 0 soldiers / 4 million civilians

Figure S4. Text of a scenario presented to subjects.

4 Moral rationality in the war dilemma

In this section we present the foundations of rational choice theory, focusing on its application to moral psychology and the war dilemma. For those readers interested in a more general treatment of the subject, we recommend Jehle & Reny's microeconomics textbook [1].

4.1 Rational choice theory in a nutshell

Rational choice theory is a formalization of folk-psychological theories of intentional behavior [2]. It aims to explain and predict the choices of decision makers who act purposefully and react to incentives.

The core of a rational choice model is *the agent.* She is an abstract representation of a real decision maker. Depending on the decision problem, the agent may represent a person, a household, a company, a political party, an army, or a non-human animal, to mention a few applications. In our application, the agent represents the moral tradeoff system.

A rational choice model has the following elements:

1. **A set of conceivable options**: This set contains all options that the agent "can conceive of," whether feasible or not at the moment of choice. Options are problem-specific, but do not depend on the prevailing incentives.

2. **A preference order**: For any two conceivable options $\mathbf{x}$ and $\mathbf{y}$, the agent can tell whether $\mathbf{x}$ is "at least as good" as $\mathbf{y}$, according to her preferences. This is written as $\mathbf{x} \succsim \mathbf{y}$. This expression is also read as "$\mathbf{x}$ is weakly preferred to $\mathbf{y}$."

3. **A feasible set**: This set contains the options that the agent perceives as available to solve her problem. A feasible set is a proper subset of the set of conceivable options.

4. **The optimization assumption**: Among all feasible options, the agent will choose one of the most preferred (there may be a tie for first place).

This minimal set of assumptions about preferences, feasible options, and optimization constitute the "axioms of rational choice."

4.2 *Homo economicus*

The axioms of rational choice theory are usually lumped together with certain auxiliary assumptions about the *content* of preferences. These assumptions include selfish preferences, love of leisure, expected utility, and geometric or exponential discounting of future utility. As a group, these assumptions loosely define the preferences of a *Homo economicus*.

Contrary to popular belief, the empirical validity of *Homo economicus* preferences is not essential to the survival of rational choice theory. If the auxiliary assumptions fail in a particular application, they can be replaced by better ones without having to revise the axioms of the theory. Rational choice theory is not about the content of an agent's preferences; it is about how the agent makes choices given whatever preferences she has.

4.3 What are preferences?

The technical definition of preference is loosely related to its colloquial meaning.

In common parlance, "to prefer" means "to like." This is a folk psychological concept that predicts a wide range of mental states, physiological reactions, and behaviors. If you tell me that you like Snickers better than Twizzlers, I will think you enjoy eating Snickers more than eating Twizzlers. I will think that you salivate more at the sight of a Snickers. I will anticipate that, given a choice between a Snickers and a Twizzlers, you will pick the Snickers.

In rational choice theory, by contrast, the word "preference" is a label attached to a mathematical object: a binary relation between the elements of a set (the set of conceivable options).

The connection between preferences (in their technical sense) and the cognitive mechanisms of decision making is mostly ignored by researchers in the field. Most are agnostic about the psychological reality of preferences: They argue that it is irrelevant whether preferences are real or fictitious. In their view, all that matters is that decision makers behave *as if* they had preferences and optimized their choices accordingly [3, 4].

This nihilistic ontology of preferences is an element of "predictionism," the prevailing epistemology of rational choice theory. Predictionism is an idiosyncratic variant of instrumentalism. It states that rational choice models are neither true nor false;

only useful or useless for predicting the choices of real decision makers [5]. Other predictions of the theory—including its axioms, which trivially predict themselves—are dismissed as immaterial. The arbitrary dismissal of some predictions separates predictionism from standard forms of instrumentalism, which maintain that all predictions of a theory—including its axioms—must be under constant empirical scrutiny.

Sweeping axioms under the rug may seem like cheating, but it's the only workable epistemology when decision makers are households or firms. Families and firms make purposeful decisions, factoring in the incentives they face, but they lack minds capable of optimization: Decisions in both types of organizations arise from internal negotiations between their members, each of whom has his own individual agenda. In the absence of a theory of internal negotiations, economists recourse to the best existing alternative, which is rational choice theory. Their hope is that, when viewed from the outside, the negotiation process approximates an optimization process, at least at the aggregate or "market" level.

But when the decision maker is a person, the claim that he can make optimal choices without a cognitive system that does the optimizing is less plausible.[4]

4.4 Moral preferences

In rational choice models, preferences can be about any kind of goods: consumer goods and services, leisure time, financial assets, prestige, mates—anything that is scarce and people desire. When rational choice theory is applied to moral psychology, preferences are about moral goods, such as human lives, improving people's welfare, or fulfilling obligations.

Regarding notation, the statement "option $\mathbf{x}$ is weakly preferred to option $\mathbf{y}$" is interpreted as "solution $\mathbf{x}$ is felt to be at least as right as solution $\mathbf{y}$." Likewise, the statement "option $\mathbf{x}$ is strictly preferred to option $\mathbf{y}$" is interpreted as "solution $\mathbf{x}$ is felt to be more right than solution $\mathbf{y}$."

Unlike orthodox rational choice theorists, who take an agnostic stance on the psychological reality of preferences, we commit to a realist ontology: Moral preferences exist temporarily in memory, represented as rightness functions.

[4]Of course, no one consciously optimizes his choices given an explicit preference order of all conceivable options; but this does not invalidate the theory. The mind is known to carry out very complex algorithms in real time (as in language and vision) without our being aware of it.

4.5 Properties of a preference order

A preference order has the following properties:

1. It is **total**: $\mathbf{x} \succsim \mathbf{y}$, or $\mathbf{y} \succsim \mathbf{x}$, or both. This means that all options can be compared with each other.

2. It is **antisymmetric**: If $\mathbf{x} \succsim \mathbf{y}$ and $\mathbf{y} \succsim \mathbf{x}$, then the agent is "indifferent" between $\mathbf{x}$ and $\mathbf{y}$. In notation, $\mathbf{x} \sim \mathbf{y}$.

3. It is **reflexive**; that is, $\mathbf{x} \succsim \mathbf{x}$. In words, every option is weakly preferred to itself (it is at least as good as itself).

4. It is **transitive**: $\mathbf{x} \succsim \mathbf{y}$ and $\mathbf{y} \succsim \mathbf{z}$ implies $\mathbf{x} \succsim \mathbf{z}$.

Transitivity is a well-known and generally uncontroversial assumption. It is usually explained with apples and oranges: "If an apple is preferred to a banana, and a banana is preferred to an orange, then an apple is preferred to an orange." The example is accurate, but leaves the wrong impression that transitivity is trivial, when in fact the opposite is true. Being rational or "consistent" (complying with the axioms of rational choice at all times) is a dauntingly complex task when options are not single goods (an apple, a banana, an orange) but bundles of goods (k apples, m bananas, and n oranges). The complexities will be apparent in section 4.18, where we will explain GARP in the context of the war dilemma.

4.6 Indifference and strict preference

A preference order creates as a by-product an indifference relation and a strict preference relation.

Indifference relation: $\mathbf{x} \sim \mathbf{y}$ if and only if $\mathbf{x} \succsim \mathbf{y}$ and $\mathbf{y} \succsim \mathbf{x}$

Strict preference relation: $\mathbf{x} \succ \mathbf{y}$ if and only if $\mathbf{y} \not\succsim \mathbf{x}$.

Both relations are transitive.

In the context of moral judgment, the statement "$\mathbf{x}$ is indifferent to $\mathbf{y}$" means "$\mathbf{x}$ is felt to be as right as $\mathbf{y}$." Likewise, the statement "$\mathbf{x}$ is strictly preferred to $\mathbf{y}$" means "$\mathbf{x}$ is felt to be more right than $\mathbf{y}$."

4.7 Utility functions

In some decision problems, the set of conceivable options is potentially infinite and boundless. The war dilemma is a case in point (imagine two billion soldiers saved and one billion civilians spared; now imagine one billion + 1).[5]

The question naturally arises as to how the mind can store and manipulate an order of preference over infinitely many options. This seems like a computational impossibility, but rational choice theorists have found a workaround: An infinite preference order can be represented by a continuous real-valued "utility function," provided that the order satisfies certain mathematical conditions.[6]

A utility function is a finite mathematical object that represents, in compact form, a preference order for all solutions that the mind can conceive of. To each conceivable solution, a utility function assigns a level of utility: a real number that serves to compare options in terms of their subjective "goodness." If two options have the same utility, they are considered equally good. Otherwise, the option with the higher utility is strictly preferred.

Formally, a utility function is written as follows:

$$u(\mathbf{x}) : X \to V \subseteq \mathbb{R},$$

where u is the function, $\mathbf{x}$ is a conceivable option, X is the set of conceivable options, and V contains the values that utility can take (which are real numbers). Like the preference order it represents, a utility function is problem-specific. But it does not depend on which options are available: The function is invariant with respect to the content of the feasible set.

A utility function gives rise to a preference order in the following manner:

If $u(\mathbf{x}) \geq u(\mathbf{y})$, then $\mathbf{x} \succsim \mathbf{y}$.

If $u(\mathbf{x}) = u(\mathbf{y})$, then $\mathbf{x} \sim \mathbf{y}$.

[5]It is an open question whether *all* quantities that are conceivable formally (in the context of mathematics and a linguistic number system; e.g., "one billion + 1") can be represented by a cognitive system that constructs preference orders (see [6]). The minds of humans and other animals represent quantities, but the format of these representations and the limits of what each computational system can represent are active areas of cognitive research.

[6]See [1, section 1.2] for an in-depth discussion of preference orders and their connection to utility functions.

If $u(\mathbf{x}) > u(\mathbf{y})$, then $\mathbf{x} \succ \mathbf{y}$.

It is tempting to associate utility with pleasure, happiness, satisfaction, or welfare; but the analogy is improper. Utility, as a mathematical object, has no intensity or degree: It tells you whether one option is better than another, not how much better it is.

When a preference order has certain mathematical properties, the corresponding utility function is continuous and doubly-differentiable. Continuity and double differentiability are convenient for modeling, because they allow the use of calculus, which simplifies constrained maximization.

4.8 Rightness functions are utility functions

In abstract terms, a rightness function can be written as follows:

$$v(\mathbf{x}, \boldsymbol{\beta}) : X \to V \subseteq \mathbb{R},$$

where v is the function; $\mathbf{x}$ is a conceivable solution; X is the set of conceivable solutions; V contains the values that rightness can take (which are real numbers); and $\boldsymbol{\beta}$ is a vector of situation-specific parameters computed by the MV subsystem, by operating on morally-laden representations of the situation. (See the main text for a model of the moral tradeoff system; MV is the component that constructs rightness functions.)

The rightness function gives rise to a rightness order of conceivable solutions, as follows:

If $v(\mathbf{x}, \boldsymbol{\beta}) = v(\mathbf{y}, \boldsymbol{\beta})$, then both solutions are felt to be equally right.

If $v(\mathbf{x}, \boldsymbol{\beta}) > v(\mathbf{y}, \boldsymbol{\beta})$, then $\mathbf{x}$ is felt to be more right than $\mathbf{y}$.

The parameters in $\boldsymbol{\beta}$ modulate the way in which the rightness function ranks solutions. For example, in the war dilemma, $\boldsymbol{\beta}$ contains the relative weights assigned to the lives of civilians and soldiers, plus other parameters having to do with elasticity of substitution and/or other aspects of preferences.

Context can affect the parameters values. For example, if the civilians had supported the war, rather than opposed it, civilian lives would probably weigh less. Likewise, if the soldiers had volunteered instead of being forcibly drafted, soldiers' lives would probably weigh less.

4.9 Testing for consistency requires auxiliary assumptions

One can always rationalize a sequence of moral judgments by saying that the subject is indifferent among all conceivable solutions, and hence all her choices were arbitrary. More formally, the rightness function $v(\mathbf{x}) = k$, where k is a constant, rationalizes any sequence of moral judgments. It follows that the moral tradeoff system hypothesis cannot be falsified in isolation (an inconvenience that extends to rational choice theory in general). Making auxiliary assumptions about the shape of the rightness function is an inescapable requirement.

4.10 Well-behavedness

In our empirical analysis, we made the auxiliary assumption that rightness functions are "well-behaved." Contrary to what the term suggests, well-behavedness is not an ethical standard that a rightness function can instantiate. Rather, well-behavedness is a set of mathematical properties that some utility functions satisfy: continuity, nonsatiation, and convex indifferent curves.[7] The meaning of these properties is mathematically involved, but it has an intuitive geometric interpretation.

Figure S5 depicts two rightness functions viewed from above: One is well-behaved, while the other is not. The points on the upper-right quadrant of the Cartesian plane (axes included) represent the set of conceivable solutions. Point (c, s) is a solution involving c civilians spared and s soldiers saved. Elevation at a given point is the rightness of the corresponding solution. All solutions that lie on the same contour line are felt equally right. The arrows indicate the direction in which rightness increases.

A well-behaved rightness function (such as the one depicted in panel a) resembles a mountain with no peak and no backside. It is nonsatiated: Starting from any solution (any point on the Cartesian plane), one can increase rightness by increasing c, s, or both quantities (mathematically speaking, the function is strictly increasing). The contour lines of a well-behaved rightness function are "indifference curves." This means that all solutions that are equally right lie on the same contour line. Viewed from the origin, well-behaved indifference curves are convex (though not necessarily strictly convex). In jargon, this means that there is "substitution" between goods.

[7]A more general form of well-behavedness assumes local nonsatiation, a less stringent mathematical requirement on the structure of the preference order.

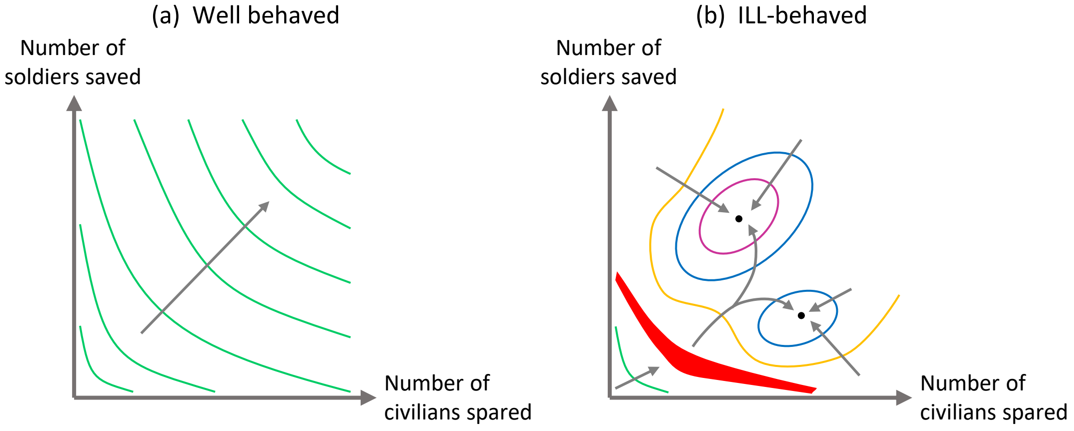

Figure S5. Two rightness functions viewed from above.

The rightness function depicted in panel *b* misbehaves in various ways. It has two peaks (marked with black dots), where it is satiated. It has more than one contour line with the same level of moral rightness (such as the blue ones), which are thus not indifference curves. Its surface has a flat "indifference zone" (highlighted in red), where the function has a constant value. And some indifference curves are not convex (for example, the orange one).

We consider nonsatiation and convex indifference curves to be psychologically plausible assumptions for the war dilemma.

Nonsatiation formalizes two intuitions: Given the alternative of saving more soldiers without increasing civilian deaths, a typical subject will take that alternative. Likewise, given the alternative of saving more civilians without increasing soldiers' deaths, a typical subject will take that alternative.

Convex indifference curves, on the other hand, formalize an intuition about the substitutability of soldiers' and civilian lives: For a subject to be indifferent between a series of alternative solutions, each additional civilian death must be compensated by saving the lives of a non-decreasing number of additional soldiers. In other words, measured in terms of civilian lives, each additional soldier life is worth the same or less than the previous one.

It should be emphasized that we do not posit that all rightness functions are well-behaved. It just seemed likely to us that, in this particular experiment, most subjects would exhibit well-behaved rightness functions.

The experimental results confirmed our guess.

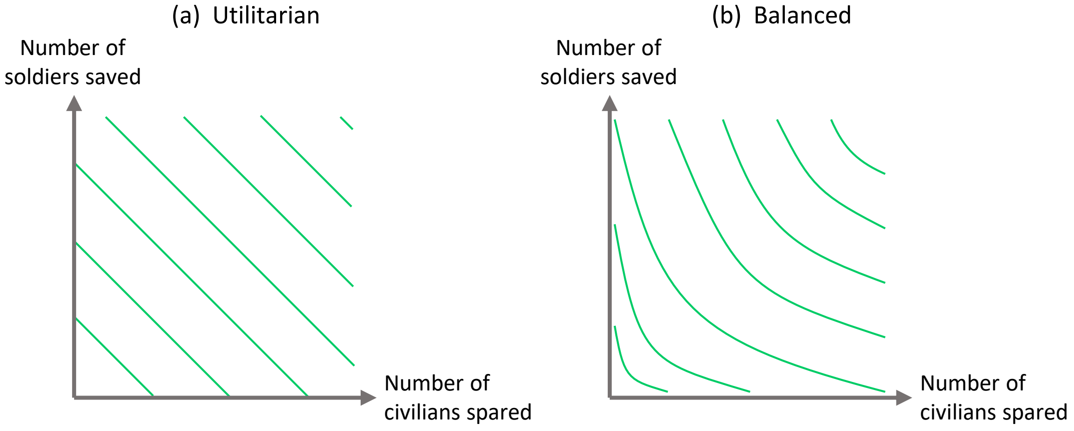

Figure S6. Two types of rightness functions.

4.11 Utilitarian and balanced rightness functions

Figure S6 depicts two rightness functions. Both are well-behaved.

The utilitarian value function (depicted in panel a) is given by $v(c,s) = c + s$. According to this formula, only the total number of survivors matters from a moral point of view. The function's indifference curves are straight lines: They have the form $s = r - c$, where parameter $r > 0$ is the level of rightness of that curve. Straight indifference curves are convex, but not strictly convex.

To illustrate one kind of rightness function that can produce compromise judgments, consider the "balanced" function in panel b (short for "striking a balance"). It has indifference curves that bend smoothly inwards. In mathematical terms, they are strictly convex to the origin. Strict convexity implies that, for the subject to remain indifferent among a series of alternative solutions, each additional civilian death must be compensated by the lives of an increasing number of additional soldiers. Put differently, each additional surviving soldier is worth less to the subject than the previous one in terms of civilian lives.

4.12 Feasible solutions to the war dilemma

The feasible set or "feasibility constraint" of a scenario is given by

$$F = \{(c,s) : s = -(S/C)c + S, \text{ where } c, s \geq 0\}.$$

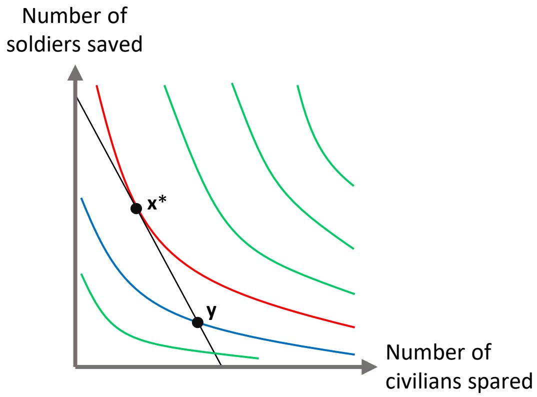

Figure S7. A balanced rightness function and a linear feasibility constraint. The optimal solution $\mathbf{x}^*$ is at the point of tangency between the constraint and the red indifference curve.

where S and S/C are parameters, and $S > C > 0$. These parameters constitute the incentives of the dilemma. S is the number of soldiers at risk of death. S/C is the efficacy of bombing: the number of soldiers saved per civilian sacrificed.

Figure S7 provides an example.

4.13 Compromises judgments as rightness maximizing choices

Figure S7 depicts a balanced rightness function. The diagonal line segment represents a feasibility constraint; imagine that all points on the line segment are available to be chosen.

Since this balanced rightness function has strictly convex indifference curves that approach, but do not intersect, the axes, the optimal solution $\mathbf{x}^* = (c^*, s^*)$ is unique and lies at the point of tangency between the feasibility constraint and an indifference curve. In consequence, the optimal solution is intermediate. More formally, when all points on the feasibility constraint are available and the rightness function is balanced, it is always the case that $c^*, s^* > 0$: That is, the optimal solution will always be a compromise.[8]

[8]For the war dilemma, feasibility constraints were discretized to the nearest million, so not all points on the line segment were available to be chosen. In this case, a person with these indifference curves might choose a mixture of compromise and extreme solutions. Imagine, counterfactually, that the feasible set included every point on the line connecting $(0, S)$ and $(C, 0)$. The optimal solution would be the point of tangency, and intermediate. But let's say the number of civilians spared at the optimum is closer to 0 than to 1M (i.e., anywhere from 1 to 499,000); when the feasible set is

As a proof, consider solution $\mathbf{y}$, which lies at the intersection between the feasibility constraint and the blue indifference curve. Observe that solutions on the blue curve have a lower level of rightness than those on the red curve. It follows that $\mathbf{y}$ is less right than $\mathbf{x}^*$. We conclude that $\mathbf{y}$, and any feasible solution other than $\mathbf{x}^*$, cannot be optimal.

In contrast, a subject who maximizes a utilitarian rightness function [i,e., $v(c, s) = c + s$] will choose the solution with the greatest possible number of survivors. In the war dilemma, the number of survivors is maximized when all soldiers are saved and no civilians are spared (from a maximum possible number of C). That is, $\mathbf{x}^* = (0, S)$.

4.14 Deontic moral values

Deontic values cannot be represented by a well-behaved rightness function, but they can be represented by a rule: "Prefer a solution that maximizes the number of civilians spared. Among solutions that maximize the number of civilians spared, prefer one that maximizes the number of soldiers saved."[9] The moral tradeoff system could construct this rule on the fly and store it temporarily in memory, like a computer stores an active program in RAM (but this is speculative).

4.15 Rational moral flip-flopping

Linear rightness functions can cause moral flip-flopping.

Consider the following example:

$$v(c, s) = \alpha c + (1 - \alpha)s,$$

where $0 < \alpha < 1$. And recall that the feasibility constraint of the war dilemma is

$$s = -\frac{S}{C}c + S,$$

where $0 \leq c \leq C$ and $0 \leq s \leq S$. This constraint implies that, if $c = 0$, then $s = S$. It also implies that, if $c = C$, then, $s = 0$.

discretized to the nearest million, an extreme solution that spares no civilians might be closer to that point than the intermediate solutions offered. A mix of compromise and extreme judgments can be produced by indifference curves with other properties as well (e.g., ones with a similar shape that do intersect the axes).

[9]Deontic values are a form of "lexicographic preference," which are notorious for not being representable by a continuous utility function.

The maximization problem can be easily solved by replacing s in the rightness function with the feasibility constraint. This reduces to:

$$v(c) = \alpha c + (1-\alpha)\left(-\frac{S}{C}c + S\right).$$

Moreover, grouping terms, we obtain:

$$v(c) = (1-\alpha)\left(\beta - \frac{S}{C}\right)c + (1-\alpha)S,$$

where $\beta = \alpha/(1-\alpha)$. Observe that β is an increasing function of α. Also, $\beta \in (0, \infty)$.

The solution to this optimization problem will depend on the sign of the coefficient accompanying c. There are three cases:

1. If $S/C > \beta$, then the coefficient will be negative, and so the optimal solution will be to minimize the value of c. Therefore, $c^* = 0$ and $s^* = S$. This means that the subject will issue a "utilitarian" judgment.

2. If $S/C < \beta$, then the coefficient will be positive, so the optimal solution will be to maximize the value of c. Therefore, $c^* = C$ and $s^* = 0$. This means that the subject will issue a "deontic" judgment.

3. If $S/C = \beta$, then the coefficient will be zero. Therefore, all feasible values of (c, s) will maximize rightness, and so the model will make no prediction. This degenerate case will occur with zero probability if S/C is a continuous random variable.

It follows that the subject will exhibit a flip-flopping response pattern, in response to changes in the incentives of the dilemma. Note that linear rightness functions are one of many types of rightness functions that can cause a flip-flopping response profile.

4.16 Revealed preferences, hand trembles, and inconsistency

As a subject makes choices, she gradually reveals preference relations, which may or may not match her true preference relations.

The simplest type of revelation occurs when the feasible set contains two options: $\mathbf{x}$ and $\mathbf{y}$. If the subject chooses $\mathbf{x}$, then "$\mathbf{x}$ is revealed weakly preferred to $\mathbf{y}$." Otherwise, we presume, the subject would have chosen $\mathbf{y}$. We denote this inference "$\mathbf{x} \succsim^R$

$\mathbf{y}$." Superscript R distinguishes revealed preferences (inferred from choices) from the subject's true, underlying preferences.

If a subject abides by the axioms of rational choice, without ever making a mistake, her revealed preferences will be accurate. Perfect rationality, in the sense of consistency, guarantees that $\mathbf{x} \succsim^R \mathbf{y}$ implies $\mathbf{x} \succsim \mathbf{y}$, $\mathbf{x} \sim^R \mathbf{y}$ implies $\mathbf{x} \sim \mathbf{y}$, and $\mathbf{x} \succ^R \mathbf{y}$ implies $\mathbf{x} \succ \mathbf{y}$.

A human, however, occasionally makes "trembling hand mistakes": unintended choices caused by clumsiness, distraction, and other forms of noise. Because of trembling hand mistakes, some revealed preference relations may be incorrect. For instance, an unintended choice could reveal $\mathbf{x} \succsim^R \mathbf{y}$, whereas in reality $\mathbf{y} \succ^R \mathbf{x}$. Contradictory revelations of this sort are called *inconsistencies*. A special case follows logically: "$\mathbf{x} \succ^R \mathbf{x}$" is an inconsistency, because $\mathbf{x} \succsim^R \mathbf{x}$ by definition.

A subject's revealed preference order grows as she makes more choices, but it can never fully encompass a boundless set of conceivable solutions (unlike true preference orders, revealed preference orders are not necessarily total). Even if a subject always makes consistent choices, her true preference order never becomes fully known.

4.17 Preference inference rules

The preference elicitation procedure that we used in this study is based on GARP. It exploits three inference rules.

The first inference rule derives from the optimization assumption:

> **Rule 1:** If a subject chooses $\mathbf{x}$, she reveals that she weakly prefers $\mathbf{x}$ to every other option in the feasible set (she reveals $\mathbf{x} \succsim^R \mathbf{y}$ for each feasible option $\mathbf{y}$).

This rule produces directly revealed preference relations; that is, relations that link options that are in the same feasible set.

The second rule derives from the transitivity assumption. It links three options in a revealed preference chain:

> **Rule 2:** $\mathbf{x} \succsim^R \mathbf{y}$ and $\mathbf{y} \succ^R \mathbf{z}$ implies $\mathbf{x} \succ^R \mathbf{z}$.

Options $\mathbf{x}$, $\mathbf{y}$, and $\mathbf{z}$ need not all be in the same feasible set. If they are, the preference revelation is direct. Otherwise the preference revelation is indirect.

The third rule derives from the nonsatiation assumption:

> **Rule 3:** If $\mathbf{x}$ is Pareto superior to $\mathbf{y}$, then $\mathbf{x} \succ^R \mathbf{y}$.

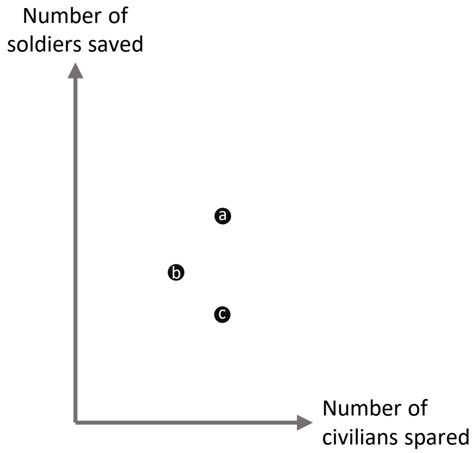

Figure S8. Solution $\mathbf{a}$ is Pareto superior to $\mathbf{b}$ and $\mathbf{c}$. Therefore, $\mathbf{a}$ is revealed to be strictly preferred to both solutions.

This rule takes the preference for Pareto superior options as *a priori* knowledge.

In the war dilemma, solution $\mathbf{x}$ is Pareto superior to solution $\mathbf{y}$ if and only if three conditions are met: (1) More soldiers are saved and/or more civilians are spared in $\mathbf{x}$ than in $\mathbf{y}$. (2) The number of civilians spared in $\mathbf{x}$ is not less than in $\mathbf{y}$. (3) The number of soldiers saved in $\mathbf{x}$ is not less than in $\mathbf{y}$.

By way of example, consider solutions $\mathbf{a}$, $\mathbf{b}$, and $\mathbf{c}$ depicted in figure S8. $\mathbf{a}$ is Pareto superior to $\mathbf{b}$, because $\mathbf{a}$ saves more soldiers and spares more civilians than $\mathbf{b}$. For this reason, we assume that the subject strictly prefers $\mathbf{a}$ to $\mathbf{b}$ (i.e., $\mathbf{a}$ is felt more right than $\mathbf{b}$). Moreover, $\mathbf{a}$ is Pareto superior to $\mathbf{c}$, because $\mathbf{a}$ and $\mathbf{c}$ spare the same number of civilian lives, but $\mathbf{a}$ saves more soldiers. Therefore, rule 3 leads us to conclude that the subject strictly prefers $\mathbf{a}$ to both $\mathbf{b}$ and $\mathbf{c}$.

On the other hand, $\mathbf{b}$ is not Pareto superior to $\mathbf{c}$, and $\mathbf{c}$ is not Pareto superior to $\mathbf{b}$, because $\mathbf{b}$ saves more soldiers but spares fewer civilians than $\mathbf{c}$. Therefore, we cannot tell *a priori* whether the subject is indifferent between $\mathbf{b}$ and $\mathbf{c}$, or strictly prefers one solution to the other.

In addition to the three inference rules, the procedure assumes $\mathbf{x} \succsim^R \mathbf{x}$. Intuitively speaking, it is taken for granted that each option is at least as good as itself.

4.18 The generalized axiom of revealed preferences

Here we will derive GARP for the particular case of the war dilemma, using the three inference rules presented in section 4.17.

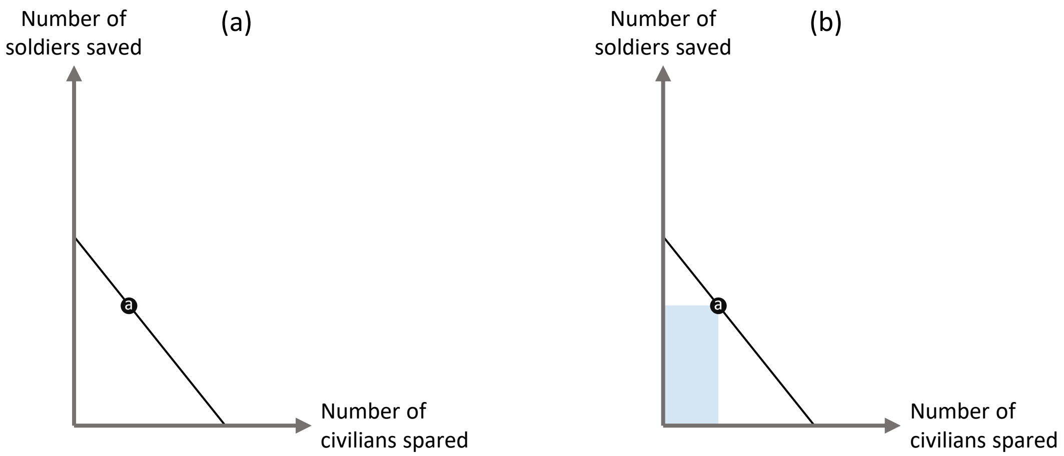

Figure S9. (a) A scenario of the war dilemma. The subject chooses solution $\mathbf{a}$. (b) For each solution $\mathbf{x}$ in the light blue rectangle, $\mathbf{a}$ is Pareto superior to $\mathbf{x}$. It follows that $\mathbf{a} \succ \mathbf{x}$ for each $\mathbf{x}$ in the rectangle (inference rule 3).

Two preliminary notes on the well-behavedness assumption: First, well-behavedness is sufficient but more stringent than necessary for GARP. Second, ill-behavedness does not invalidate the moral tradeoff system hypothesis, because ill-behaved rightness functions lead to rational judgments, though not necessarily of the GARP-respecting kind. Depending on their choices, subjects who exhibit ill-behaved rightness functions may be misclassified by GARP as inconsistent, a possibility that biases the test against the hypothesis.

We begin by analyzing the simplest case: a subject facing two scenarios.

First, the subject faces the scenario depicted in figure S9a. She chooses solution $\mathbf{a}$ from the feasibility constraint. This choice reveals that she weakly prefers $\mathbf{a}$ to every other solution on the constraint, and strictly prefers $\mathbf{a}$ to every solution below the constraint. This inference is explained step by step in figures S9b to S10b.

As a corollary, we obtain a fourth inference rule:

> **Rule 4:** If the subject chooses solution $\mathbf{y}$ from a feasibility constraint, then, for each solution $\mathbf{x}$ below the constraint, $\mathbf{y} \succ^R \mathbf{x}$.

Next, the subject faces the scenario depicted in figure S11a. This scenario has a different feasibility constraint, with a steeper slope and a higher intercept. The steeper slope (a higher value of S/C) means that the death of one civilian saves a greater number of soldiers. The higher intercept (a higher value of S) means that more soldiers are at risk of death.

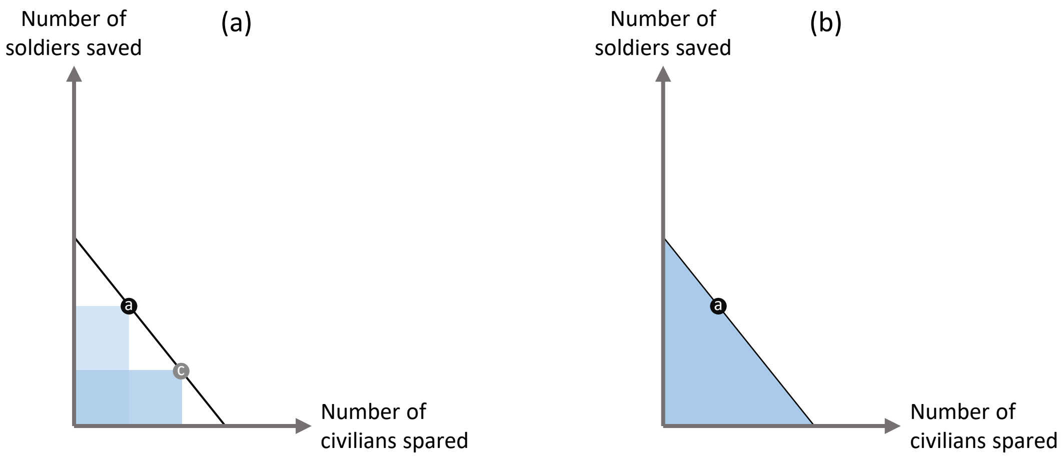

Figure S10. (a) Solution $\mathbf{c}$ was feasible, but the subject did not choose it. It follows that $\mathbf{a} \succsim^R \mathbf{c}$ (inference rule 1). Moreover, for each solution $\mathbf{x}$ in the darker blue rectangle, $\mathbf{c}$ is Pareto superior to $\mathbf{x}$; hence, $\mathbf{c} \succ^R \mathbf{x}$ (inference rule 3). From $\mathbf{a} \succsim^R \mathbf{c}$ and $\mathbf{c} \succ^R \mathbf{x}$, it follows that $\mathbf{a} \succ^R \mathbf{x}$ for each $\mathbf{x}$ in the darker blue rectangle (inference rule 2). (b) The previous argument extends to all other unchosen solutions on the feasibility constraint. Each of these solutions has a corresponding blue rectangle of Pareto inferior solutions. The rectangles overlap, creating a blue triangle below the constraint. We conclude that, for each solution $\mathbf{x}$ below the feasibility constraint, $\mathbf{a} \succ^R \mathbf{x}$.

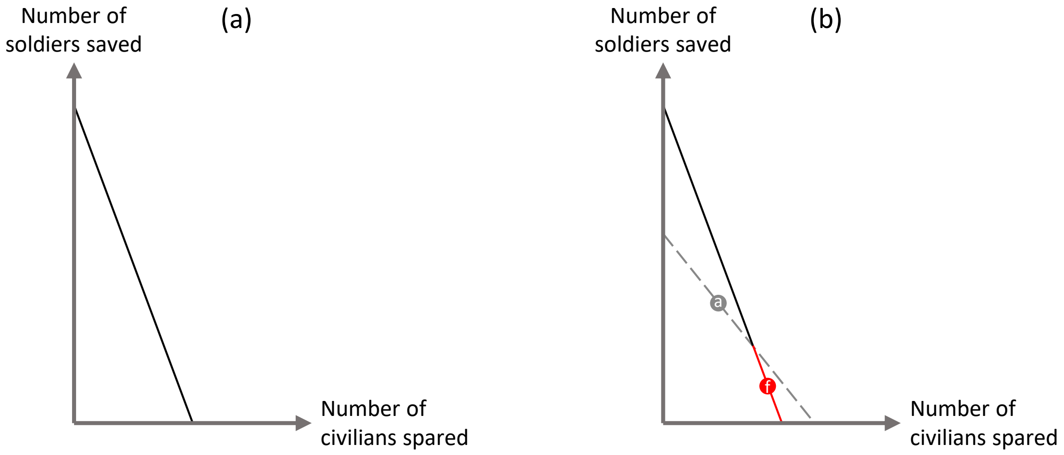

Figure S11. (a) The subject faces a second scenario. (b) GARP predicts that a morally rational subject would not choose a solution on the red segment. To see why, suppose that she chooses $\mathbf{f}$. This choice reveals that $\mathbf{f} \succ^R \mathbf{a}$, because $\mathbf{a}$ is below the current feasibility constraint (inference rule 4). On the other hand, $\mathbf{f}$ is below the previous feasibility constraint (the dashed line). It follows that $\mathbf{a} \succ^R \mathbf{f}$ (inference rule 4), which contradicts $\mathbf{f} \succ^R \mathbf{a}$. This inconsistency is a GARP violation. Note: There is a special case in which $\mathbf{a}$ is at the intersection of the two constraints. In that case, $\mathbf{f} \succsim^R \mathbf{a}$, because $\mathbf{a}$ is an unchosen solution on the second constraint (inference rule 1). This revelation also contradicts $\mathbf{a} \succ^R \mathbf{f}$.

What solutions could the subject rationally choose?

Figure S12 shows the feasibility constraint divided into two segments, one green and one red. GARP predicts that a morally rational subject would choose a solution on the green segment. We prove this graphically in figure S12.

There are also cases in which the second choice will not cause inconsistencies or "GARP violations," regardless of what the subject chose first. This is proved in figure S13.

More generally, GARP allows for arbitrarily long sequences of scenarios. The following inference rules link n solutions, within and across scenarios, in chains of revealed preference relations:

> **Rule 5a:** If $\mathbf{x}_1 \succsim^R \mathbf{x}_2 \succsim^R \ldots \succsim^R \mathbf{x}_n$ and $\mathbf{x}_n \succsim^R \mathbf{x}_{n+1}$, then $\mathbf{x}_1 \succsim^R \mathbf{x}_2 \succsim^R \ldots \succsim^R \mathbf{x}_n \succsim^R \mathbf{x}_{n+1}$.
>
> **Rule 5b:** If $\mathbf{x}_1 \succsim^R \mathbf{x}_2 \succsim^R \ldots \succsim^R \mathbf{x}_n$ and $\mathbf{x}_n \succ^R \mathbf{x}_{n+1}$, then $\mathbf{x}_1 \succsim^R \mathbf{x}_2 \succsim^R \ldots \succsim^R \mathbf{x}_n \succ^R \mathbf{x}_{n+1}$.

These rules are generalizations of rule 2.

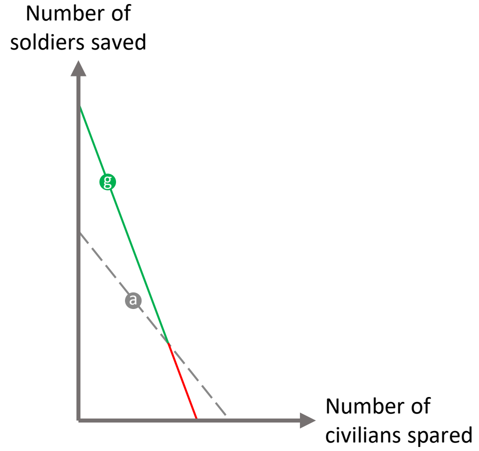

Figure S12. A morally rational subject would choose a solution on the green segment. To see why, suppose she chooses $\mathbf{g}$. This reveals that $\mathbf{g} \succ^R \mathbf{a}$, because $\mathbf{a}$ is below the second constraint (inference rule 4). On the other hand, because $\mathbf{g}$ is above the first constraint (the dashed line) it was not available to be chosen when the subject chose $\mathbf{a}$ from the first feasibility constraint. So there are no reasons to infer $\mathbf{a} \succsim^R \mathbf{g}$ (rules 1 or 4 cannot be used). Hence, no inconsistency is found.

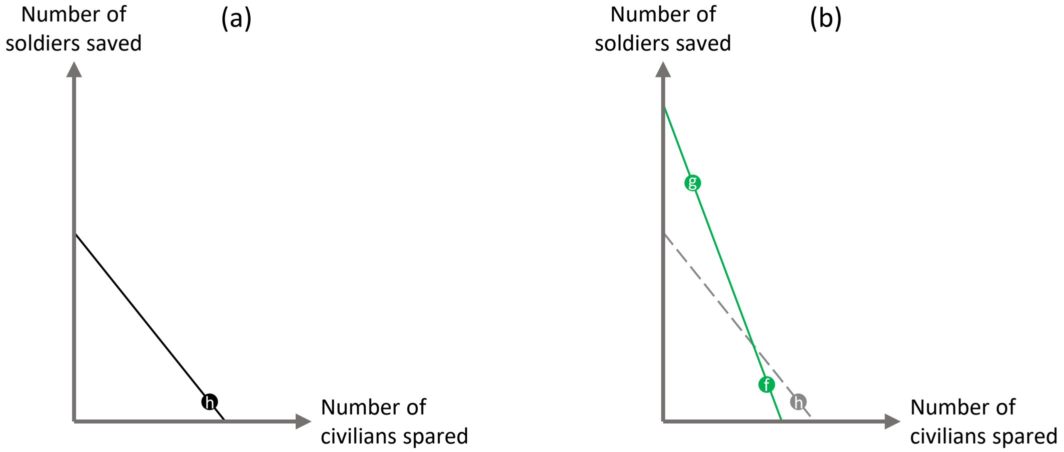

Figure S13. (a) A different subject chooses $\mathbf{h}$ from the first feasibility constraint. (b) In the second scenario, whatever choice he makes (such as $\mathbf{f}$ or $\mathbf{g}$) would be morally rational. This is because $\mathbf{h}$ is to the right of the second constraint, so the subject's second choice does not reveal a preference relation between $\mathbf{h}$ and any choice on the second constraint (rules 1 or 4 cannot be used).

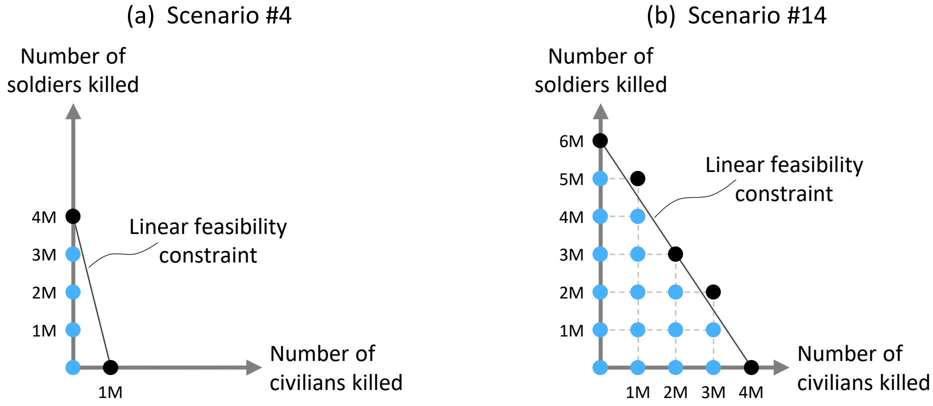

Figure S14. Black markers represent discretized feasible solutions. Blue markers represent solutions that are Pareto inferior to at least one feasible solution. (a) $S = 4$ and $S/C = 4$. This scenario offers two extreme solutions. (b) $S = 6$ and $S/C = 1.5$. This scenario offers two extreme and three intermediate solutions.

Due to rules 5a and 5b, a single choice may reveal a large number of preference relations. These relations may link solutions that never appear in the same feasibility constraint, or even in the same scenario. Cascades of GARP violations can occur as a consequence.

4.19 Discretized solutions

Counting GARP violations requires discretizing the solution space. A discretized feasible set approximates a linear feasibility constraint, such as the ones shown in figure S14. Solutions in the discretized feasible set fall approximately on the line (they are rounded to the nearest million). A Pareto inferior solution is inferior to at least one member of the feasible set.

4.20 Counting GARP violations

To count GARP violations, we used the same method as Andreoni & Miller [7].

Consider, for example, a subject that faces scenarios 13, 17, and 19. Table S4 shows the feasible set and Pareto inferior solutions of these scenarios.

First, the subject faces scenario 13 and chooses solution $(2, 2)$. Since $(1, 4)$ also appears in the feasible set, her choice reveals $(2, 2) \succsim^R (1, 4)$ (inference rule 1). Moreover, since $(1, 4)$ is Pareto superior to $(1, 3)$, we assume that $(1, 4) \succ^R (1, 3)$ (inference

Table S4. Three scenarios of the war dilemma

Scenario #	Soldiers at risk (S)	Soldiers saved for each civilian sacrificed (S / C)	Alternatives (civil. sacrificed, sold. dead)	Feasible set (civil. spared, sold. saved)	Pareto inferior solutions (civil. spared, sold. saved)
13	6	2.00	(0, 6) (1, 4) (2,2) (3,0)	(3, 0) (2, 2) (1, 4) (0, 6)	(0, 0) (1, 0) (2, 0) (0, 1) (1, 1) (2, 1) (0, 2) (1, 2) (0, 3) (1, 3) (0, 4) (0, 5)
17	7	3.50	(0, 7) (1, 4) (2, 0)	(2, 0) (1, 3) (0, 7)	(0, 0) (1, 0) (0, 1) (1, 1) (0, 2) (1, 2) (0, 3) (0, 4) (0, 5) (0, 6)
19	7	1.75	(0, 7) (1, 6) (2, 4) (3, 2) (4, 0)	(4, 0) (3, 1) (2, 3) (1, 5) (0, 7)	(0, 0) (1, 0) (2, 0) (3, 0) (0, 1) (1, 1) (2, 1) (0, 2) (1, 2) (2, 2) (0, 3) (1, 3) (0, 4) (1, 4) (0, 5) (0, 6)

Note: All quantities in millions of lives.

rule 3). Applying rule 5b to both revelations, we infer:

$$(2,2) \succsim^R (1,4) \succ^R (1,3).$$

Next, the subject faces scenario 17 and chooses solution $(1,3)$. Since $(0,7)$ also appears in the feasible set, her choice reveals $(1,3) \succsim^R (0,7)$ (inference rule 1); and we previously inferred that $(2,2) \succsim^R (1,4) \succ^R (1,3)$. Applying rule 5a to both revelations, we infer:

$$(2,2) \succsim^R (1,4) \succ^R (1,3) \succsim^R (0,7). \tag{1}$$

Lastly, the subject faces scenario 19 and chooses solution $(0,7)$. Since $(2,3)$ also appears in the feasible set, her choice reveals $(0,7) \succsim^R (2,3)$. Moreover, since $(2,3)$ is Pareto superior to $(2,2)$, we assume that $(2,3) \succ^R (2,2)$. Applying rule 5b to both revelations, we infer:

$$(0,7) \succsim^R (2,3) \succ^R (2,2) \tag{2}$$

Using inference rules 5a and 5b we can link preference chains (1) and (2), as follows:

$$(2,2) \succsim^R (1,4) \succ^R (1,3) \succsim^R (0,7) \succsim^R (2,3) \succ^R (2,2).$$

We have thus found a "preference cycle."

The above preference cycle entails that, for each pair of solutions $\mathbf{x}$ and $\mathbf{y}$ in the set $\{(2,2),(1,4),(1,3),(0,7),(2,3)\}$, it is the case that "$\mathbf{x} \succ^R \mathbf{y}$ and $\mathbf{y} \succsim^R \mathbf{x}$" (which includes as a particular case "$\mathbf{x} \succ^R \mathbf{x}$"). Each of these contradictions is a GARP violation. Since there are five solutions in the cycle, the number of GARP violations created by the cycle is $\binom{5}{2} + 5 = 15$.

Note that the preference cycle that we have detected is one of several caused by the subject's third choice. You can find the remaining cycles using the same procedure that we have illustrated here.

5 The representative agent's rightness function

We conjectured that the representative agent maximizes a CES rightness function:

$$u((c,s),\beta(k)) = \alpha(k)^{\frac{1}{\sigma(k)}} c^{\frac{\sigma(k)-1}{\sigma(k)}} + [1-\alpha(k)^{\frac{1}{\sigma(k)}}] s^{\frac{\sigma(k)-1}{\sigma(k)}},$$

where c and s are the numbers of surviving civilians and soldiers, $k = \{\text{SW}, \text{BU}, \text{CW}\}$ represents the frame, and $\beta(k) = [\alpha(k), \sigma(k)]$ is a vector of parameters whose values can be frame-dependent.

Parameter α is the weight of civilian lives on moral rightness. Possible values of α range from 0 to 1, where $\alpha > 0.5$ indicates more weight on the lives of civilians relative to soldiers, and $\alpha = 0.5$ indicates equal weight.

Parameter $\sigma > 0$ is the elasticity of substitution between civilian and soldiers' lives. Elasticity of substitution determines the degree of curvature of the indifference curves. It has a behavioral implication: The higher the value of σ, the more sensitive the agent's responses are to changes in S/C.

We estimated the representative agent's rightness function indirectly, by fitting her optimal response function (recall that her response in each scenario is the average for all subjects). A optimal response function gives the number of soldiers to be saved, for given values of S, S/C, and k (the frame). In logarithms,

$$ln(s^*) = ln \frac{[1-\alpha(k)]S}{1-\alpha(k)+\alpha(k)(S/C)^{1-\sigma(k)}} + \varepsilon,$$

where s^* is the optimal response, and ε is an error term.

We performed a nonlinear least-squares regression with $n = 21 \times 3 = 63$ observations (21 obs. per condition). Its results are reported in Table S5.

The estimated values of α are significantly different in each condition. The representative agent puts the most weight on civilian lives ($\alpha = 0.80$) when they opposed the war but soldiers were willing to fight for their country. When civilians and soldiers were both unwilling participants, the agent also put more weight on the lives of civilians ($\alpha = 0.61$), but less than in the previous case. Only when civilians supported the war and soldiers were unwilling draftees, did the agent weight civilians and soldiers similarly ($\alpha = 0.49$).

Table S5. Nonlinear least-squares regression
of the representative agent's optimal response function

				Num. obs.	63	
				R-squared	0.9975	
				Adj. R-squared	0.9972	
				Root MSE	0.0569	
				Res. dev.	-186.61	

ln(*s*)	Coef.	Robust std. err	t-stat.	P-value	[99% conf. inter.]	
α(SW)	0.80	0.0064	124.23	0.000	0.78	0.81
α(BU)	0.61	0.0076	79.99	0.000	0.59	0.63
α(CW)	0.49	0.0062	79.37	0.000	0.47	0.51
σ(SW)	1.88	0.0369	50.86	0.000	1.78	1.97
σ(BU)	1.99	0.0367	54.11	0.000	1.89	2.08
σ(CW)	1.98	0.0384	51.53	0.000	1.88	2.08

References

[1] Geoffrey A. Jehle and Philip J. Reny. *Advanced Microeconomic Theory*. 3rd. New York: Pearson, 2011. ISBN: 9780273731917.

[2] Alexander Rosenberg. *Economics: Mathematical Politics or Science of Diminishing Returns*. Chicago: University of Chicago Press, 1992. ISBN: 9780226727233.

[3] Milton Friedman. "The methodology of positive economics". In: *Essays in Positive Economics*. Chicago: University of Chicago Press, 1953, pp. 3–43. ISBN: 0226264033.

[4] Faruk Gul and Wolfgang Pesendorfer. "The case for mindless economics". In: *The Foundations of Positive and Normative Economics: A Handbook*. Ed. by Andrew Caplin and Andrew Schotter. New York: Oxford University Press, 2008, pp. 3–39. ISBN: 9780195328318.

[5] Daniel M. Hausman. "Economic methodology in a nutshell". In: *Journal of Economic Perspectives* 3.2 (1989), pp. 115–127. DOI: 10.1257/jep.3.2.115.

[6] Christina Boyce-Jacino et al. "Large numbers cause magnitude neglect: The case of government expenditures". In: *Proceedings of the National Academy of Sciences* 119.28 (2022), e2203037119. DOI: 10.1073/pnas.220303711.

[7] James Andreoni and John Miller. “Giving according to GARP: An experimental test of the consistency of preferences for altruism”. In: *Econometrica* 70.2 (2002), pp. 737–753. DOI: 10.1111/1468-0262.00302.
